# A tunable and reversible thermo-inducible bio-switch for streptomycetes

**DOI:** 10.1093/nar/gkae1236

**Published:** 2024-12-19

**Authors:** Lanxin Lv, Shuo Liu, Yudie Fu, Yuxin Zhang, Meiyan Wang, Jiahe Sun, Yi Wang, Yinhua Lu, Guoqing Niu

**Affiliations:** College of Agronomy and Biotechnology, Southwest University, No.2, Tiansheng Road, Beibei District, Chongqing 400715, China; College of Agronomy and Biotechnology, Southwest University, No.2, Tiansheng Road, Beibei District, Chongqing 400715, China; College of Agronomy and Biotechnology, Southwest University, No.2, Tiansheng Road, Beibei District, Chongqing 400715, China; College of Agronomy and Biotechnology, Southwest University, No.2, Tiansheng Road, Beibei District, Chongqing 400715, China; College of Agronomy and Biotechnology, Southwest University, No.2, Tiansheng Road, Beibei District, Chongqing 400715, China; Integrative Science Center of Germplasm Creation in Western China (CHONGQING) Science City, Biological Science Research Center, Southwest University, No.2, Tiansheng Road, Beibei District, Chongqing 400715, China; Integrative Science Center of Germplasm Creation in Western China (CHONGQING) Science City, Biological Science Research Center, Southwest University, No.2, Tiansheng Road, Beibei District, Chongqing 400715, China; College of Life Sciences, Shanghai Normal University, No.100 Guilin Road, Xuhui District, Shanghai 200234, China; College of Agronomy and Biotechnology, Southwest University, No.2, Tiansheng Road, Beibei District, Chongqing 400715, China

## Abstract

Programmable control of bacterial gene expression holds great significance for both applied and academic research. This is particularly true for *Streptomyces*, a genus of Gram-positive bacteria and major producers of prodigious natural products. Despite that a few inducible regulatory systems have been developed for use in *Streptomyces*, there is an increasing pursuit to augment the toolkit of high-performance induction systems. We herein report a robust and reversible thermo-inducible bio-switch, designated as StrepT-switch. This bio-switch enables tunable and reversible control of gene expression using physiological temperatures as stimulation inputs. It has been proven successful in highly efficient CRISPR/Cas9-mediated genome engineering, as well as programmable control of antibiotic production and morphological differentiation. The versatility of the device is also demonstrated by thermal induction of a site-specific relaxase ZouA for overproduction of actinorhodin, a blue pigmented polyketide antibiotic. This study showcases the exploration a temperature-sensing module and exemplifies its versatility for programmable control of various target genes in *Streptomyces* species.

## Graphical Abstract

**Graphical Abstract:**
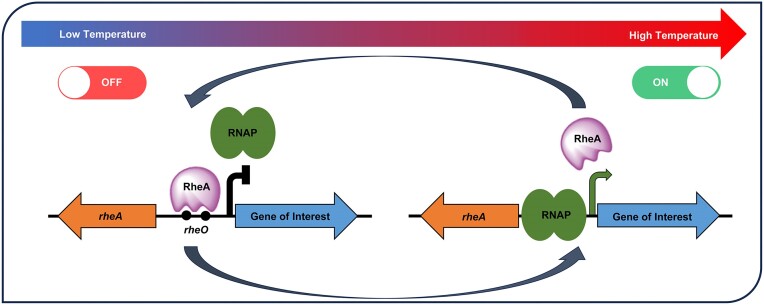

## Introduction

*Streptomyces* is a unique genus within the phylum Actinobacteria of Gram-positive bacteria. Distinct from most bacteria, *Streptomyces* species display a multicellular life cycle strikingly similar to that of filamentous fungi. Notably, they are renowned for their prolific capability to produce numerous bioactive natural products (1,2). Over the past few decades, a plethora of genetic tools have been developed for *Streptomyces*. Among them, only a small number of induction systems are available for use in *Streptomyces* (3). Typically, they are based upon transcriptional regulators that can sense a specific chemical signal and then activate the expression of target genes. Examples include regulatory systems that respond to a variety of chemical stimuli, such as antibiotics, sugars and other chemicals (4). However, the current inducible systems have certain limitations. For instance, antibiotic inducers are accompanied by toxicity (5–7), and sugars are utilized as nutrients (4,8,9). Most significantly, all chemical inducers lack the property of reversibility. To address these issues, it is imperative to devise high-performance induction systems by exploring alternative stimuli. In a previous study, a temperature-inducible expression system was established based upon a mutated repressor TraR and its target promoter from the plasmid pSN22 of *Streptomyces nigrifaciens*. This system has been used for inducible production of malate dehydrogenase (MDH) of *Thermus flavus* in *Streptomyces lividans* (10). However, the applications of this inducible system in metabolic pathway engineering have yet to be investigated.

When exposed to a sudden temperature upshift, microorganisms respond by rapidly inducing the expression of various heat shock proteins (HSPs), including molecular chaperones and proteases. In bacteria, transcriptional regulation of heat-shock genes can be either positive or negative. Positive regulation exploits alternative sigma factors to redirect RNA polymerase to heat shock gene promoters, while negative regulation relies on dedicated transcriptional repressors (11). In *Streptomyces* species, the synthesis of different HSPs is under the control of different regulatory networks (11). It is noteworthy that the regulator RheA represses the expression of the *hsp18* gene. The *hsp18* gene is located upstream and in the opposite orientation to the repressor, and it encodes a small HSP (12). The RheA binding region covers the -10 box and transcription start site (TSS) of the *rheA* promoter, indicating that RheA synthesis is negatively autoregulated. At lower temperatures, RheA exerts its regulatory function by binding directly to its operator (*rheO*) embedded within the intergenic region between *rheA* and *hsp18*. At higher temperatures, RheA senses temperature changes and relieves its repression on *hsp18* expression. Intriguingly, the temperature-induced derepression by RheA is reversible (12).

To fulfill the need for robust, tunable and reversible control of gene expression, we created two highly responsive thermo-inducible genetic systems, referred to as *TRS01* and *TRS02*. The regulatory systems are generated based upon the thermo-sensing RheA repressor and the *hsp18* promoter. Both *TRS01* and *TRS02* exhibit a high level of induced reporter activity and almost no leaky expression in the model organism *Streptomyces albidoflavus* J1074. *TRS02* enables tunable and reversible control of target gene expression with alternating temperature stimuli. Thus, the thermo-inducible bio-switch is designated as StrepT-switch. The device has been proven successful for highly efficient CRISPR/Cas9-mediated genome engineering, as well as programmable control of antibiotic production and morphological differentiation. The application of this system has also been demonstrated by thermal induction of ZouA-mediated actinorhodin overproduction. This study demonstrates the exploration a temperature-sensing module and exemplifies the versatility of this system for programmable control of the expression of various genes of interest. Our study enriches the toolkit of inducible regulatory systems that are crucial for fine-tuning gene expression in *Streptomyces*, a major source of medically important natural products.

## Materials and methods

### Bacterial strains and culture conditions

Bacterial strains, plasmids and primers used in this study are presented in Supplementary Tables S1, S2 and S3 of the Supplementary Material, respectively. *Escherichia coli* DH5α was employed as a general host strain for routine subcloning. *E. coli* ET12567 (pUZ8002) was utilized as a host for transferring DNA from *E. coli* to *Streptomyces* by intergeneric conjugation (13). *E. coli* BW25113 (pIJ790) was used for the construction of recombinant plasmids *via* λ-Red-mediated recombination technology (14). For general purposes, *Streptomyces* strains were grown on mannitol soya flour (MS) agar or in yeast extract-malt extract (YEME) liquid medium (13). Other culture media are specified in the following sections. Unless otherwise stated, *Streptomyces* strains were cultivated at 28°C, and *E. coli* strains were maintained at 37°C. When necessary, antibiotics were applied at the following concentrations: 100 μg/mL ampicillin, 100 μg/mL apramycin, 50 μg/mL kanamycin, 100 μg/mL hygromycin and 25 μg/mL chloramphenicol in Luria-Bertani (LB) medium for *E. coli*; 50 μg/mL apramycin, 50 μg/mL hygromycin and 25 μg/mL nalidixic acid in MS medium for *Streptomyces*.

### Construction of reporter plasmids

For the construction of pSET152::*RS01*-*neo*, a 959 bp fragment encompassing the coding region of *rheA* with the intergenic region between *rheA* and *hsp18* was PCR-amplified from the genomic DNA of *S. albidoflavus* J1074 using primer pair RS01 F/R. The *t0* transcriptional terminator was PCR-amplified with primer pair t0 F/R from pIJ8660 (15). Subsequently, the terminator was assembled with the 959 bp fragment by employing overlap extension PCR with primers t0 F and RS01 R. The resulting amplicon was cut with *Xba*I. The promoter-less neomycin phosphotransferase gene (*neo*) originating from transposon Tn5 was PCR-amplified with primer pair neo orf F/R from pUC119::*neo* and cut with *Eco*RI. Prior to PCR amplification, the neo orf F primer was phosphorylated with T4 polynucleotide kinase to facilitate subsequent ligation. The two fragments were ligated together with *Xba*I/*Eco*RI double-digested pSET152 in a three-piece ligation reaction to generate pSET152::*RS01*-*neo*. A similar strategy was employed for the construction of pSET152::*TRS01*-*neo*, except that the native promoter of *rheA* was replaced by the constitutive *hrdB* promoter and the *tfd* transcriptional terminator. The generation of pSET152::*TRS02*-*neo* was accomplished by inserting an additional *rheO* immediately after the TSS of *hsp18* promoter in pSET152::*TRS01*-*neo*.

For the construction of pSET152::*TRS02*-*gusA*, the coding region of *gusA* was PCR-amplified with primer pair gusA F/R from pSET152-*P_hrdB_*-*gusA* (16). The resulting amplicon was then utilized to replace the *neo* cassette in pSET152::*TRS02*-*neo*, thereby generating pSET152::*TRS02*-*gusA*. The construction of pSET152::*TRS02*-*egfp* was accomplished by a similar strategy, except that the coding region of *egfp* was PCR-amplified with primer pair egfp F/R from pIJ8660 (15).

### Construction of CRISPR/Cas9 plasmids

For the construction of pKCcas9::StrepT-switch-d*dptABC*, StrepT-switch was used to replace the *tipA* promoter in pKCcas9dO (17), leading to the generation of pKCcas9-StrepT-switch. Next, two fragments, covering appropriately 1.0 kb of homologous upstream and downstream of *dptABC* within the daptomycin biosynthetic gene cluster, were amplified by PCR from the genomic DNA of *S. roseosporus* NRRL 15998 using primer pairs dpt Up F/R and dpt Dn F/R. The amplicons were assembled together and inserted into pKCcas9-StrepT-switch *via* a homologous recombination technology following the manufacturer's instructions (ClonExpress MultiS One Step Cloning Kit, Vazyme Biotech Co., Ltd.). A similar strategy was utilized for the construction of pKCcas9::kasO*-KI StrepT-switch except that the constitutive kasO* promoter was inserted between the upstream and downstream homologous fragments.

For the construction of pSET152::StrepT-switch-*dCas9*-*actI1*, StrepT-switch was used to replace Cel-RS2 in pSET152::*Cel*-*RS2*-*dCas9*-*actI1*(4). For the construction of pSET152::StrepT-switch- *dCas9*-*ftsZ*, the sgRNA that specifically targets *ftsZ* was PCR-amplified with primer pair *ftsZ* sgRNA F/R. The amplicon was digested with *Spe*I and *Eco*RI, and then used to replace the sgRNA that specifically targets *actI*-*ORF1* within pSET152::StrepT-switch-*dCas9*-*actI1*. The sgRNA and donor sequences were presented in Supplementary Table S4 of the Supplementary Material.

### Construction of ZouA plasmids

Plasmids for ZouA-mediated DNA amplification were constructed as described previously (18) with some modifications. In brief, two fragments containing *RsA* and *RsB* were PCR-amplified from the genomic DNA of *S. kanamyceticus* CGMCC 4.1441 using primer pairs RsA F/R and RsB F/R, respectively. Subsequently, the two fragments were assembled with the *neo* cassette and then used to capture the *act* gene cluster *via* λ-Red-mediated recombination (14). The resulting pIJ10500::*act* containing the *act* gene cluster flanked by *RsA* at the left border and the *neo* cassette and *RsB* at the right border. For the construction of pSET152::StrepT-switch-*zouA*, the coding region of *zouA* was PCR-amplified from the genomic DNA of *S. kanamyceticus* CGMCC 4.1441 with primer pair ZouA F/R. The resulting amplicon was utilized to replace the *neo* cassette in pSET152::*TRS02*-*neo* to generate pSET152::StrepT-switch-*zouA*.

### Intergeneric conjugation and analysis of exconjugants

Intergeneric conjugation between *E. coli* and *Streptomyces* was performed essentially as described previously (13). Briefly, *E. coli* ET12567 (pUZ8002) containing the conjugative plasmid was grown in LB with the appropriate antibiotics until the optical density at 600 nm (OD_600_) reached 0.5. The cells were washed twice with LB and resuspended in a final volume of 500 μL of LB. In the meantime, the *Streptomyces* spores were washed twice with double-distilled water and suspended in 500 μL 2 × YT broth at a concentration of ∼0.5 × 10^8^ per mL. The *Streptomyces* spores were heat-shocked at 50°C for 10 min to serve as recipients. Donor and recipient cells were mixed and spread on MS agar plates containing 10 mM MgCl_2_ and grown for 16–18 h at 28°C. The plates were overlaid with 1 mL water containing nalidixic acid and appropriate selective antibiotics as required and incubated at 28°C for an additional 5–7 days. For CRISPR/Cas9-mediated target knock-in or knock-out, the number of exconjugants were then directly counted on MS agar plates containing apramycin. Each conjugation was spread on three plates, and three independent experiments were conducted to calculate the exconjugants. For CRISPR interference (CRISPRi), the exconjugants were directly selected on MS agar plates containing apramycin.

### Assessment of promoter strength with kanamycin resistance gene as reporter

The evaluation of promoter strength was carried out essentially as described previously (4). In brief, spores of recombinant strains were harvested and resuspended in double-distilled water. The optical density of the spore suspension was measured at 450 nm (OD_450_). Each suspension was adjusted to an OD_450_ of 1.0. Serial 10-fold dilutions of each sample were prepared and spotted on minimal medium (MM) with mannitol (13) as the sole carbon source. For assessing promoter strength, a gradually increasing concentration of kanamycin was supplied to evaluate kanamycin resistance. The plates were cultivated at different temperatures for 5 days before being photographed.

### GUS assays

For agar plate-based assays, *S. albidoflavus* strains were streaked on R2 agar plates (13) containing 30 mg/mL 5-bromo-4-chloro-3-indolyl-β-D-glucuronide (X-gluc). For strains of *S. coelicolor* and *S. lividans*, spores of recombinant strains were harvested and resuspended in double-distilled water. The optical density of the spore suspension was measured at 450 nm and then normalized to a value of 1.0. A total of 10 μL spore suspension was spotted on R2 agar plates containing 30 mg/mL X-gluc. All plates were maintained at different temperatures and photographed after 4 days of incubation. Spectrophotometric GUS assays were performed as described previously (19).

### Determination of cell growth

Cell growth of *Streptomyces* strains was measured by a simplified diphenylamine colorimetric method as described previously (20). In brief, spores of *Streptomyces* strains were collected and inoculated into 10 mL liquid TSB. After being incubated for 48 h as a seed culture, 0.5 mL of the seed culture was transferred into 50 mL of R5A (21). Samples (0.5 mL) were harvested at an interval of 24 h, followed by centrifugation at 12, 000 g for 5 min. Cell pellets were dissolved in the diphenylamine reaction buffer (diphenylamine 1.5 g, glacial acetic acid 100 mL, concentrated sulfuric acid 1.5 mL, 1.6% aqueous acetaldehyde 1 mL) and vortexed for 1 min. The reaction mixtures were incubated at 60°C for 1 h and then centrifuged at 12, 000 g for 1 min. The OD_595_ of the supernatants was assayed using the multimode Varioskan LUX microplate reader (Thermo Scientific).

### Overexpression and purification of RheA

The coding region of *rheA* was PCR-amplified from the genomic DNA of *S. albidoflavus* J1074 using primer pair rheA orf F/R. The PCR product was digested with *Nde*I and *Eco*RI and then inserted into the corresponding sites of pET28a to generate pET28a::*rheA*. The recombinant plasmid was confirmed by DNA sequencing and subsequently introduced into *E. coli* BL21(DE3) for expression. RheA protein was overexpressed as a N-terminal His-tagged fusion protein, and purified using nickel-nitrilotriacetic acid (Ni-NTA) agarose chromatography according to the manufacturer's instructions (GE Healthcare). The purified protein was quantified following the protocol of a bicinchoninic acid (BCA) protein assay kit (Sangon Biotech Co., Ltd.).

### Electrophoretic mobility shift assays (EMSAs)

The electrophoretic mobility shift assays (EMSAs) were performed essentially as described (22). Briefly, probes of *RS01* and *RS02* were PCR-amplified with primer pair rheO F/R from pSET152::*TRS01*-*neo* and pSET152::*TRS02*-*neo*, respectively. The probes were incubated with various concentrations of RheA in 20 μL of reaction buffer containing 20 mM Tris-HCl (pH 7.5), 1 mM dithiothreitol (DTT), 5 mM MgCl_2_, 0.5 mg/ml bovine serum albumin (BSA), and 5% glycerol. The *hrdB* promoter was used as a negative control. After incubation at 30°C for 25 min, protein-bound DNA and free DNA were separated by electrophoresis on nondenaturing 4.5% (wt/vol) polyacrylamide gels with a running buffer containing 45 mM Tris-HCl (pH 8.0), 45 mM boric acid, and 1 mM EDTA. Gels were stained with the fluorescent dye ethidium bromide and imaged by a Bio-Rad GelDoc 2000 system.

### Light microscopy

Sterile coverslips were inserted at an angle of ∼45° into MM agar plates. Spore suspensions of *Streptomyces venezuelae* ATCC 10712 and its derivatives were prepared and 2 μL of each spore suspension was pipetted along the line where the surface of the coverslips met the medium. After incubation at 28°C or 37°C for 3 days, the coverslips were withdrawn and fixed with formaldehyde for 30 s. After washing with double-distilled water, the coverslips were then covered with crystal violet staining solution. After staining for one minute, the crystal violet solution was removed by washing with double-distilled water. The coverslips were left to dry under a heating lamp, mounted on glass slides, and fixed with a few drops of 50% glycerol prior to observation under a light microscope of Nikon ECLIPSE Ei.

### Confocal laser-scanning fluorescence microscopy analysis

Spores were inoculated into liquid YEME media and incubated at 28°C on a rotary shaker (220 rpm) for 48 h as seed culture. Subsequently, 1 mL of seed culture was transferred into 50 mL of R5A liquid media, with the incubation temperatures being alternated between lower and higher temperatures. After cultivation for every 12 h, 1 mL culture was withdrawn and the cells were collected by centrifugation. All samples were washed with double-distilled water to remove residual culture media and then resuspended in 10% glycerol. The suspensions were then observed under Leica SP8 confocal laser-scanning microscope at excitation wavelengths of 488 nm and emission wavelengths of 495∼530 nm. Images were merged using the Leica confocal software.

### Measurement of actinorhodin production

The quantification of actinorhodin was performed essentially as described previously (23). In brief, *S. coelicolor* M145 derivatives were grown in 50 mL of R5MS at 28°C. After cultivation, 1 mL of culture was withdrawn at different time intervals and treated with KOH (1 N final concentration). The titer of actinorhodin was then calculated by measuring the absorbance at 640 nm.

### Assessment of the thermal bio-switch in *E. coli*

All strains were inoculated into liquid LB media and cultivated overnight at 37°C. The overnight cultures were diluted 1:100 in 3 mL LB supplemented with 100 μg/mL ampicillin and 10 μg/mL kanamycin. When necessary, isopropyl β-D-1-thiogalactopyranoside (IPTG) was supplied at a concentration of 0.5 mM. The culture was incubated at 28°C or 37°C with shaking at 220 rpm for 12 h, and then cellular growth was determined by measuring the optical density at 600 nm.

### Data analyses

All data are average values of independent experiments calculated with their standard deviations by using GraphPad Prism 9. Statistical analyses involving multiple comparisons were performed with one-way ANOVA by IBM SPSS Statistics 27. Genome sequencing was conducted by Shanghai Majorbio Bio-pharm Technology Co., Ltd. by using PacBio RS II and Illumina HiSeq platforms. The data were analyzed on the free online platform of Majorbio Cloud Platform (www.majorbio.com). Minimap2 was employed for the alignment of whole-genome sequences, leveraging its efficiency and accuracy in handling large genomic datasets (24). NGenomeSyn was utilized for visualization, enabling a comprehensive representation of genomic synteny and structural variations within the genome (25).

## Results

### Construction of high-performance thermo-inducible modules

**Figure 1.**
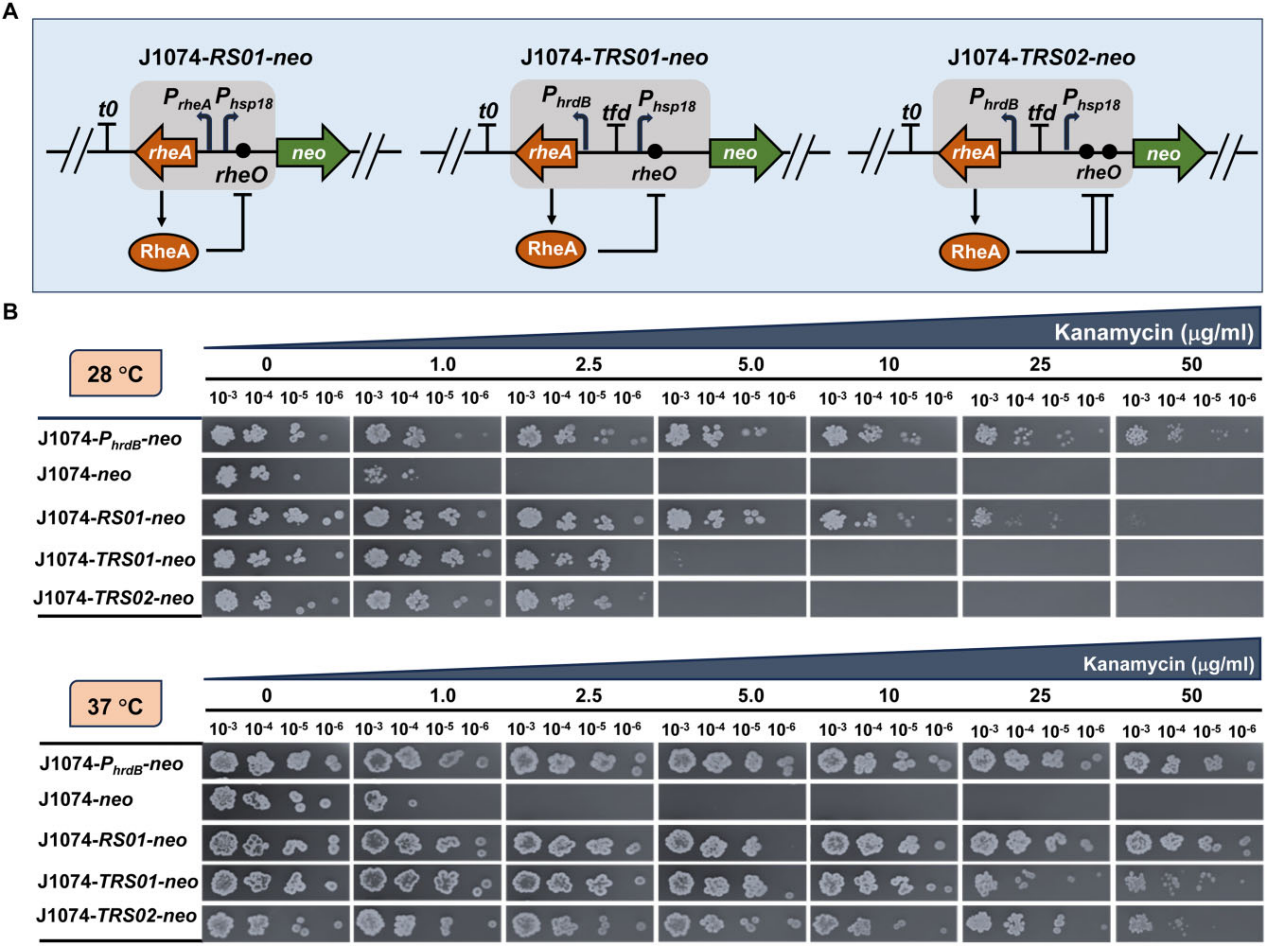
Assessment of the thermo-inducible modules. (**A**) Construction and optimization of thermo-inducible modules in *S. albidoflavus* J1074. The thermo-inducible modules were employed to drive the expression of the neomycin phosphotransferase gene (*neo*). The *t0* terminator was included to prevent interference from unwanted transcriptional readthrough. (**B**) Evaluation of the thermo-inducible modules. The overall performance of each regulatory system was assessed based on titrations of kanamycin resistances using MM agar plates cultivated at 28°C and 37°C. J1074-*neo* containing the promoter-less *neo* was included to serve as negative control. Spores of recombinant strains were harvested and resuspended in double-distilled water. The spore suspension was normalized to 1.0 at OD_450_ and serially diluted. Diluted spores were spotted on MM agar plates supplemented with increasing concentrations of kanamycin. Photographs were taken of the top of the plate after 5 days of growth. Data are representative of three independent experiments.

Inducible gene expression systems typically consist of two main components: a transcriptional regulator (TF) and a cognate target promoter harbouring TF-specific DNA-binding sequences. As mentioned earlier, the thermo-sensing repressor RheA exerts its regulatory function by regulating *hsp18* expression. To examine the distribution of RheA-like repressors in representative *Streptomyces* species, the amino acid sequence of RheA from *S. albidoflavus* J1074, previously *Streptomyces albus* J1074 (26), was used as a query for a BLASTp search to retrieve RheA orthologues from the National Center of Biotechnology Information (NCBI) nonredundant (NR) database. The results showed that the RheA repressor of *S. albidoflavus* J1074 (RheA_Sal_) shares high identities with its orthologues from model organisms, such as *S. venezuelae* ATCC 100712 (RheA_Sve_) and *S. avermitilis* MA-4680 (RheA_Sav_) (Supplementary Figure S1). Furthermore, the HSP18 of *S. albidoflavus* J1074 (HSP18_Sal_) shares high identities with its orthologues from *S. venezuelae* ATCC 100712 (HSP18_Sve_), *S. avermitilis* MA-4680 (HSP18_Sav_), and *S. scabies* 87.22 (HSP18_Ssc_) (Supplementary Figure S2). It is noteworthy that no orthologues of HSP18 were retrieved from *S. coelicolor* A3(2) and its phylogenetically closely related species *S. lividans* 66, while no orthologue of RheA was retrieved from *S. coelicolor* A3(2), *S. lividans* 66, and *S. scabies* 87.22. To construct thermo-inducible modules, the coding region of *rheA* along with the noncoding intergenic region between *rheA* and *hsp18* was assembled with the promoter-less neomycin phosphotransferase gene (*neo*). It is worth noting that the promoters of *rheA* and *hsp18* are located in a divergent orientation, and the RheA DNA-binding sequences (RheA operator, *rheO*) are embedded within the intergenic region (Figure 1A and Supplementary Figure S3). The construct was transferred into *S. albidoflavus* J1074 to obtain J1074-*RS01*-*neo*. For comparison, J1074-*neo* and J1074-*P_hrdB_*-*neo* were included to serve as negative control and positive control, respectively (Figure 1A). J1074-*neo* contains the promoter-less *neo* cassette, while J1074-*P_hrdB_*-*neo* contains the *neo* cassette driven by the constitutive *hrdB* promoter (4). J1074-*RS01*-*neo* was then cultivated, along with J1074-*neo* and J1074-*P_hrdB_*-*neo*, on minimal medium (MM) agar plates supplemented with increasing concentrations of kanamycin. When cultivated at 28°C or 37°C, J1074-*neo* exhibited resistance to kanamycin at a concentration of 1.0 μg/mL, yet it became sensitive when the kanamycin concentration was 2.5 μg/mL (Figure 1B). This phenomenon is consistent with our previous findings that *S. albidoflavus* J1074 exhibited a low level of intrinsic kanamycin resistance (4). When cultivated at 28°C, J1074-*RS01*-*neo* exhibited kanamycin resistance up to 25 μg/mL, suggesting that the native *hsp18* promoter exhibited a relatively high level of leaky expression (Figure 1B). To mitigate leakiness, *TRS01* was generated by replacing the native promoter of *rheA* with the constitutive *hrdB* promoter, and the *tfd* terminator was included to avoid interference from unwanted transcriptional readthrough (Supplementary Figure S3). Additionally, an extra *rheO* was inserted immediately after the TSS of *hsp18* promoter in *TRS01* to generate *TRS02* (Supplementary Figure S3). Transferring the two constructs into *S. albidoflavus* J1074 generated J1074-*TRS01*-*neo* and J1074-*TRS02*-*neo*, respectively (Figure 1A). When cultivated at 28°C, both J1074-*TRS01*-*neo* and J1074-*TRS02*-*neo* can grow on MM agar plates containing 2.5 μg/mL kanamycin. However, both strains are unable to tolerate 5.0 μg/mL or higher kanamycin concentrations (Figure 1B), suggesting that a repressor driven by a strong promoter is an effective way to minimize leaky expression. When cultivated at 37°C, J1074-*TRS01*-*neo* and J1074-*TRS02*-*neo* exhibited a high level of expression similar to that of the constitutive *hrdB* promoter, with kanamycin resistance up to 50 μg/mL (Figure 1B). Next, electrophoretic mobility shift assays (EMSAs) were conducted to examine protein-DNA interactions. For this purpose, recombinant RheA protein (∼25.4 kDa, calculated mass) was purified from *E. coli* BL21 (DE3) harboring pET28a::*rheA* (Supplementary Figure S4). Interestingly, one shift band was observed for the original *RS01* probe in a RheA concentration dependent manner, while two shifted bands for the *RS02* probe with two copies of *rheO* were noted at a lower RheA protein concentration. With an increase in protein concentration, the upper band clearly increased accompanied by the disappearance of the lower band (Supplementary Figure S5). Taken together, the results suggest that *TRS01* and *TRS02* exhibit tight modulation at 28°C and a high level of induced gene expression at 37°C in *S. albidoflavus* J1074.

### Tuning the bio-switch within the physiological range of temperatures

**Figure 2.**
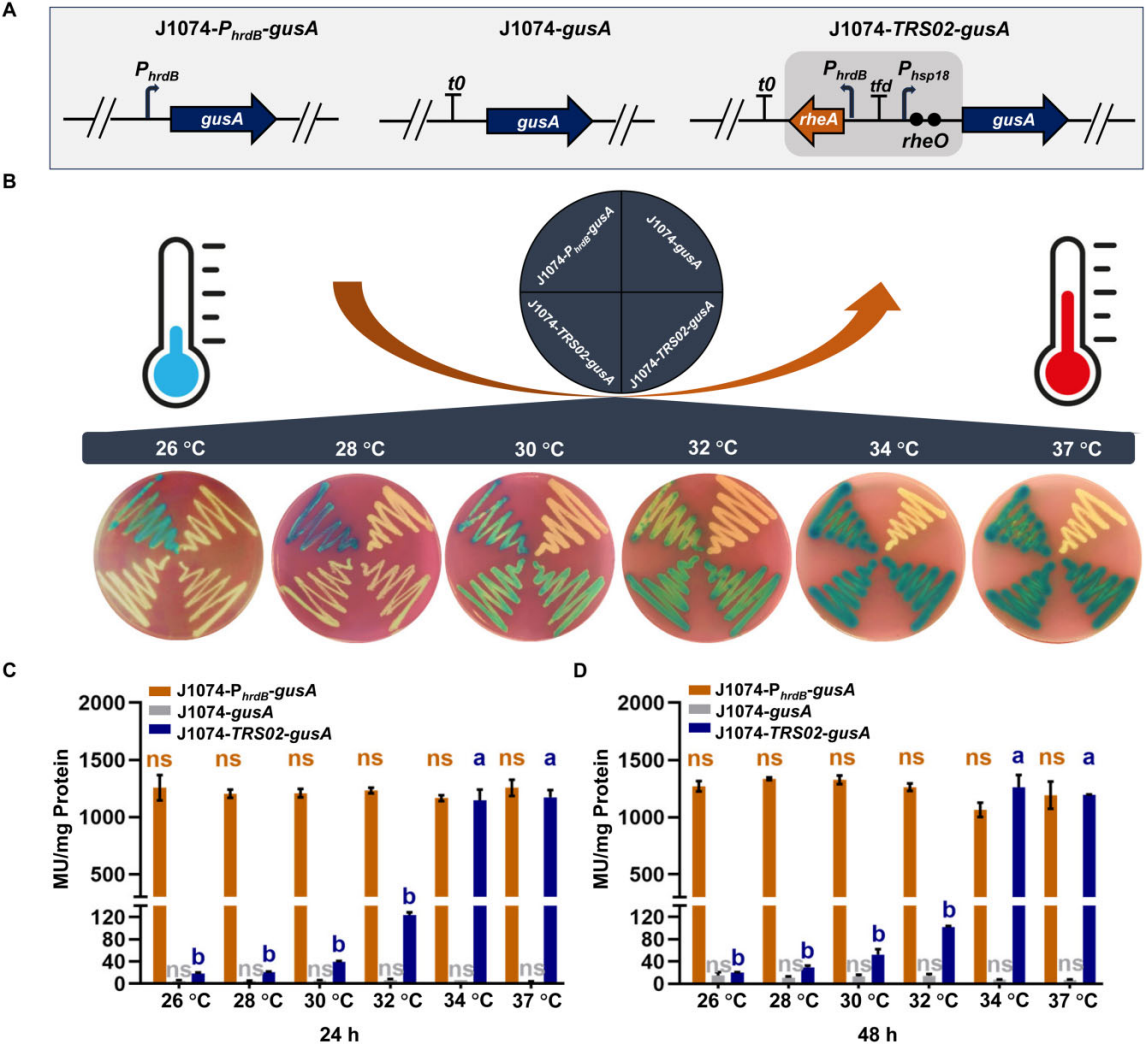
Thermal inducible expression of the reporter gene *gusA*. (**A**) Constitutive and inducible modules for *gusA* expression in *S. albidoflavus* J1074. The *gusA* gene was placed under the control of the constitutive *hrdB* promoter or the inducible regulatory system *TRS02*. A promoter-less *gusA* was included to serve as negative control. (**B**) GusA production in uninduced and induced states. The strains were cultivated on R5A agar plates at the indicated incubation temperatures. The photograph was taken from the bottom of the plate after 5 days of incubation. The representative image of three independent experiments with similar results is shown. (**C** and **D**) GusA activities were quantified after being grown for 24 and 48 h. All strains were cultivated in 50 ml of R5A liquid media. Error bars show standard deviations. Mean values with the same superscript letters are not significantly different, while those with different superscript letters are significantly different. NS: not significant.

To examine the dynamic range of the thermo-sensing modules, *TRS02* was selected to drive the expression of the reporter gene *gusA*, which encodes the β-glucuronidase enzyme (Figure 2A). For comparison, the promoter-less *gusA* and *gusA* driven by the constitutive *hrdB* promoter were included to serve as negative control and positive control, respectively (16). The resulting strains J1074-*TRS02*-*gusA*, J1074-*P_hrdB_*-*gusA* and J1074-*gusA* were cultivated on R2 agar plates for visualization of the characteristic blue color of GusA activity. Six different temperatures, covering a physiological range from 26 to 37°C, were used for cultivating these recombinant strains. An increase in cultivation temperature stimulates *gusA* expression, which in turn leads to a gradual increase in blue color intensity. A vivid blue color was observed when J1074-*TRS02*-*gusA* was incubated at 34°C and 37°C (Figure 2A). The color intensity of J1074-*TRS02*-*gusA* is similar to that of the positive control J1074-*P_hrdB_*-*gusA*. To quantify GusA activity, the strains were inoculated in liquid R5A and cultivated at six different temperatures. A gradual induction of GusA activity was observed with an increase in the cultivation temperature, and a full induction of *gusA* expression was observed at 37°C when cultivated for 24 h or 34°C when cultivated for 48 h (Figure 2C). The regulatory system shows sharp thermal transitions with a dynamic range of ∼62-fold induction. To examine bacterial growth, J1074-*TRS02*-*gusA*, J1074-*P_hrdB_*-*gusA* and J1074-*gusA* were cultivated in R5A liquid media for biomass measurements. The results showed that the three strains had comparable growth rates and biomasses at the tested temperatures (Supplementary Figure S6).

To investigate the performance of *TRS02* in other *Streptomyces* species, the construct was also transferred into the model organisms *S. coelicolor* M1146 and *S. lividans* TK24 to obtain M1146-*TRS02*-*gusA* and TK24-*TRS02*-*gusA*, respectively. An obvious induction of GusA activity was observed with M1146-*TRS02*-*gusA* (Supplementary Figure S7A). It should be noted that the strain exhibited a low level of leaky expression as judged by the light blue color of colonies at lower incubation temperatures. TK24-*TRS02*-*gusA* exhibited a high level of induced GusA activity with no leaky expression (Supplementary Figure S7B). Thus, we established a high-performance thermo-inducible system with low leakiness and high maximal expression. The results suggested that *TRS02* provides a switch-like control of gene expression with temperature as the stimulation input, and the temperature-sensing module is functional in three model *Streptomyces* species.

### The thermal bio-switch enables reversible control of gene expression

**Figure 3.**
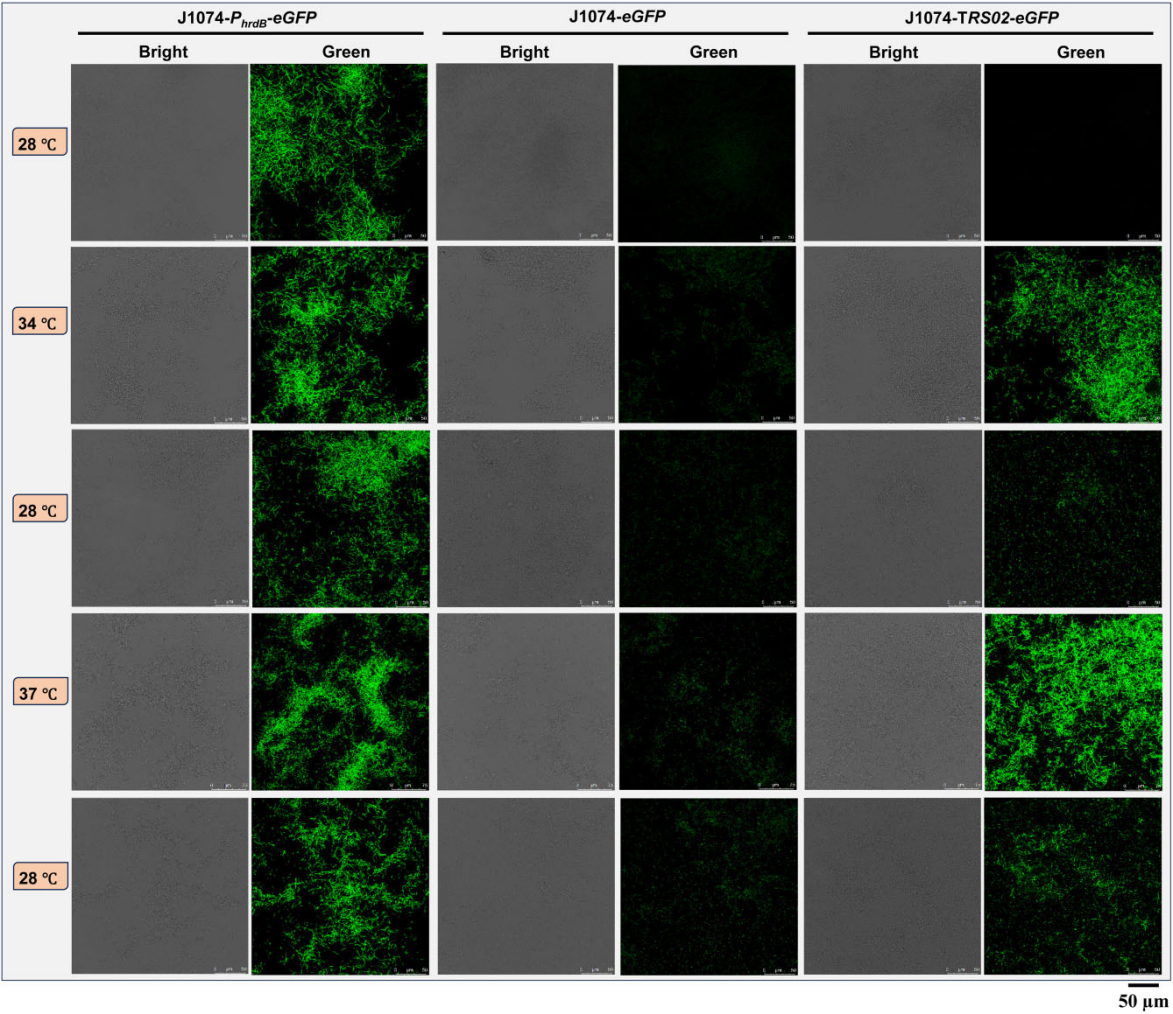
Switch-testing of reversible reporter responses. The constitutive *hrdB* promoter and *TRS02* were used to drive the expression of eGFP in *S. albidoflavus* J1074. A promoter-less *eGFP* was included to serve as negative control. All strains were cultivated in liquid R5A with incubation temperatures switched back and forth between lower and higher temperatures as indicated. The strains were cultivated for 12 h at each incubation temperature.

As mentioned earlier, existing induction systems have certain limitations. The main disadvantage is the lack of reversibility associated with chemical inducible systems. In terms of reversibility, temperature has an advantage over chemical inducers, as it can be easily adjusted. Thus, *TRS02* was used to drive the expression of enhanced green fluorescent protein (eGFP). The resulting J1074-*TRS02*-*eGFP* was cultivated in liquid R5A with incubation temperatures alternating between different temperatures. No eGFP expression was detected when the culture was initially maintained at 28°C for 12 h. An obvious eGFP expression was observed after the culture was shifted to 34°C (Figure 3). Reversibility can be achieved when the culture was switched back to 28°C, followed by fluorescence activation at 37°C and then fluorescence quenching at 28°C (Figure 3). It is noteworthy that residual fluorescent signals were observed with J1074-*TRS02*-*eGFP* in the second and third round cultivations at 28°C. Considering J1074*-eGFP*, a derivative of *S. albidoflavus* J1074 containing a promoter-less eGFP, also displayed low intensity fluorescent signals, we speculate that the fluorescent signals in J1074-*TRS02*-*eGFP* may arise from a combination of background signals and the residual eGFP left from the preceding activation event. Our results demonstrated that the thermal bio-switch, designated as StrepT-switch thereafter, enables reversible control of gene expression.

### The bio-switch facilitates highly efficient CRISPR/Cas9-mediated genome engineering

**Figure 4.**
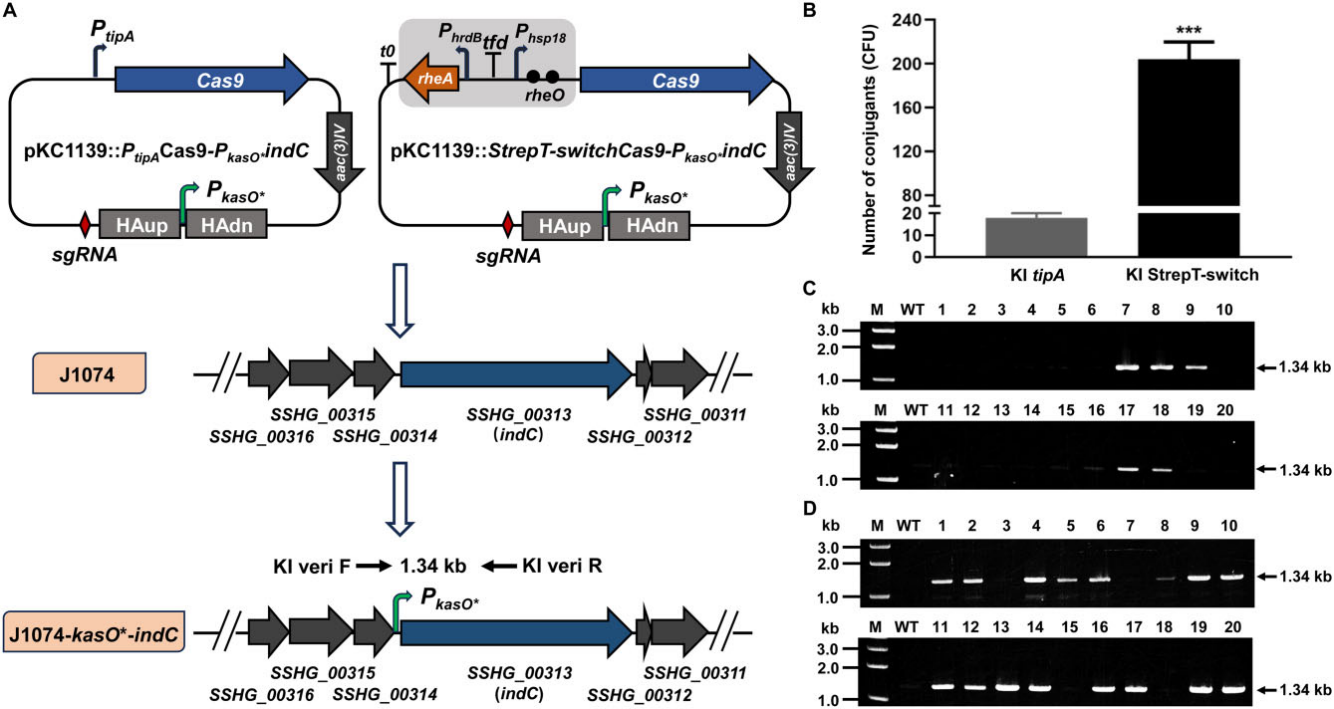
Thermal inducible CRISPR/Cas9-mediated target knock-in. (**A**) CRISPR/Cas9-mediated knock-in of the kasO* promoter preceding the coding region of *SSHG_00 313* (*indC*) in *S. albidoflavus* J1074. The *tipA* promoter or StrepT-switch was employed to drive the expression of *Cas9*. (**B**) Comparison of transformation efficiency between Cas9 driven by the *tipA* promoter (KI *tipA*-Cas9) and Cas9 driven by the StrepT-switch (KI StrepT-switch-Cas9). Asterisks are used to denote statistical significance, where the *P* value is <0.001. (**C**and**D**) Verification of transformants by PCR amplifications. The expected size of PCR amplicons is as indicated. For KI *tipA*-Cas9, all twenty obtained transformants (**1**–**20**) were utilized for PCR amplifications. For KI StrepT-switch-Cas9, 20 transformants (**1**–**20**) were randomly selected for DNA extractions and subsequent PCR amplifications. ‘kb’ represents kilobase, and ‘M’ denotes DNA Ladder. The templated from *S. albidoflavus* J1074 (WT) was included to serve as a negative control.

It is well documented that CRISPR/Cas9 shows high toxicity to the host, thus limiting its application in many *Streptomyces* strains (27). To demonstrate the utility of our bio-switch, StrepT-switch was used for flexible control of the CRISPR/Cas9-mediated genome editing tools. First, StrepT-switch was employed for CRISPR/Cas9-mediated target knock-in to activate the expression of *indC*. The *indC* gene was identified within a small gene cluster encoding the blue pigment indigoidine (28). Under routine laboratory conditions, the gene cluster remained silent in *S. albidoflavus* J1074 as no pigment formation was observed. A previous study found that substitution of the native *indC* promoter with constitutive promoter led to the activation of the gene cluster (28). For this purpose, the *tipA* promoter of *Cas9* was replaced with StrepT-switch for knock-in of the constitutive kasO* promoter preceding the coding region of *indC* (Figure 4A). When introduced into *S. albidoflavus* J1074, ∼18 exconjugants were observed on MS agar plates with the construct containing *tipA* promoter-driven *Cas9* (Figure 4B). This observation is in accordance with the fact that the *tipA* promoter exhibits a high level of leaky expression even in the absence of the thiostrepton inducer. To determine the efficiency of Cas9-based editing, 20 exconjugants were randomly picked from three independent experiments and then passaged onto MS agar plates. Subsequently, PCR amplifications with genomic DNA as the template were carried out to verify the target knock-in. The results showed that the efficiency of Cas9 for knocking-in the target gene was 25% (Figure 4C). Authenticity of the five PCR amplicons were further confirmed by DNA sequencing (Supplementary Figure S8A). In contrast, ∼204 exconjugants were obtained with the construct containing StrepT-switch-driven *Cas9*, indicating that StrepT-switch can ensure that *Cas9* is almost not expressed and its toxicity is avoided when the transformants are maintained at 28°C. After obtaining the transformants, 20 exconjugants were randomly selected from three independent experiments and then passaged onto MS agar plates. Next, the plates were incubated at 34°C to activate *Cas9* expression for CRISPR/Cas9-mediated target knock-in. Similarly, PCR amplifications with genomic DNA as the template were performed to verify the target knock-in. The results revealed that the efficiency of Cas9 for knocking-in the target gene was 80% (Figure 4D). Similarly, eight PCR amplicons were randomly selected for DNA sequencing. The results showed that these amplicons exhibited authentic base sequences (Supplementary Figure S8B). A similar strategy was used for CRISPR/Cas9-mediated target knock-out. An obvious high efficiency was observed with the knock-out of ∼39.5 kb of the daptomycin gene cluster from the chromosome of *Streptomyces roseosporus* (Supplementary Figure S9). The results showed that StrepT-switch improved the DNA transformation efficiency by reducing the toxicity of *Cas9* at lower temperature, and thus greatly increased the chance to obtain CRISPR/Cas9-mediated knock-in or knock-out mutants.

### Programmable control of antibiotic production and morphological differentiation *via* thermo-inducible CRISPRi

**Figure 5.**
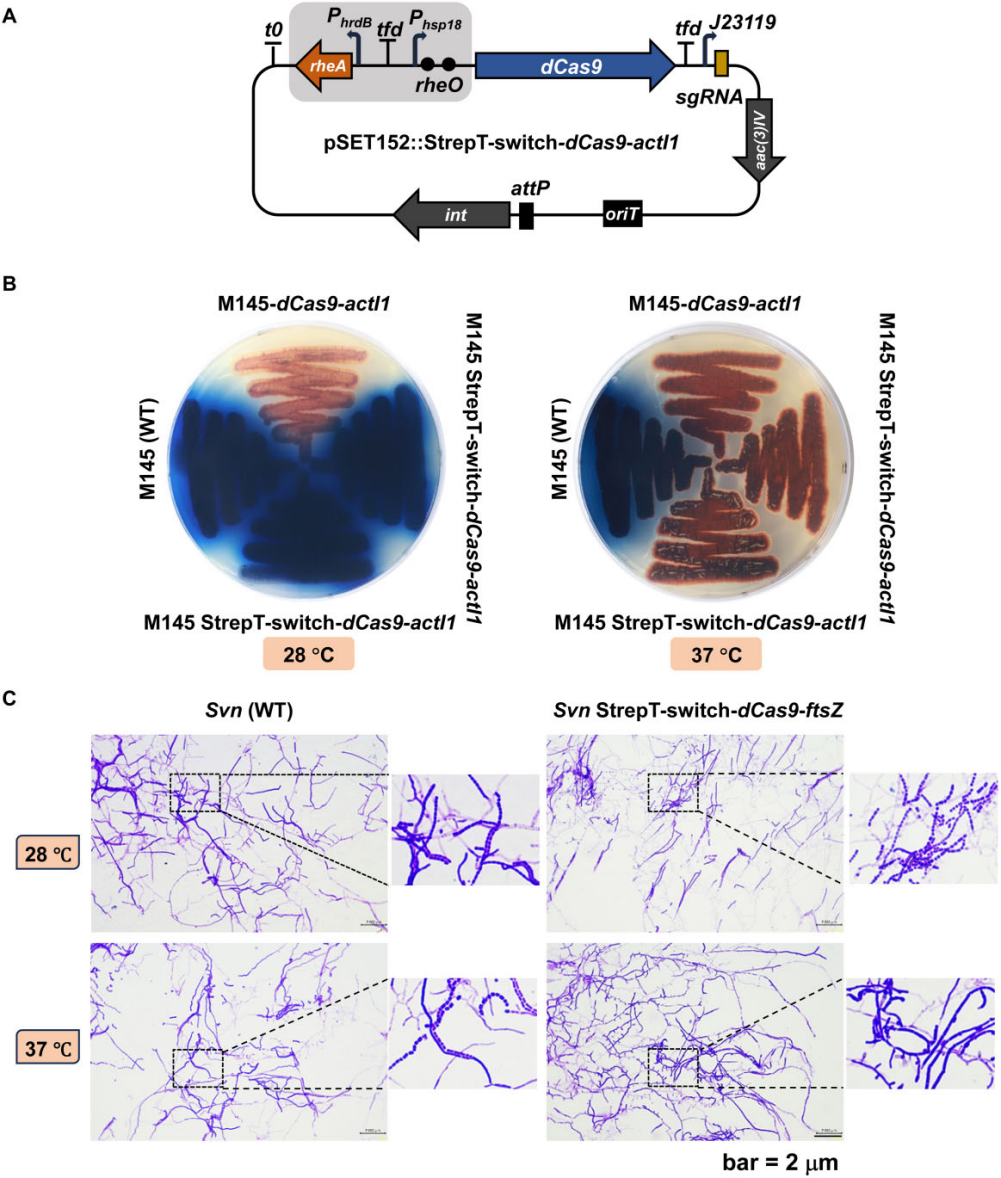
Thermal inducible CRISPRi-mediated programmable control of genes involved in antibiotic production and morphological differentiation. (**A**) Thermal inducible CRISPRi-mediated gene repression targeting *actI-ORF1* in *S. coelicolor*. (**B**) The interference of ACT production by CRISPRi depends on the incubation temperature. *S. coelicolor* strains were cultivated on R5MS agar plates at either 28°C or 37°C. The photograph was taken from the bottom of the plate after 5 days of incubation. The representative image of three independent experiments with similar results is shown. (**C**) Thermal inducible CRISPRi-mediated gene repression targeting *ftsZ* in *S. venezuelae*.

To demonstrate the utility of our thermal bio-switch, StrepT-switch was employed to repress gene expression *via* CRISPR interference (CRISPRi) in a programmable manner. In previous studies, the *dCas9* gene, a catalytically dead variant of *Streptococcus pyogenes* Cas9, was controlled either by the thiostrepton-inducible *tipA* promoter or the constitutive ermE* promoter (4,29,30). For programmable control of gene repression, a tight regulatory expression of *dCas9* is highly preferred. To this end, pSET152::*dCas9*-*actI1* was selected as it has been used for efficient repression of actinorhodin (ACT) production by targeting *actI-ORF1* (29). Thus, the expression of *dCas9* was subjected to the control of StrepT-switch rather than the constitutive ermE* promoter (Figure 5A). The resulting construct was then introduced into *S. coelicolor* M145 to obtain M145-StrepT-switch-*dCas9*-*actI1*. For comparison, M145-*dCas9*-*actI1* was included to serve as a positive control. Unlike *S. coelicolor* M145, which could produce the blue pigment ACT and the red pigment undecylprodigiosins (RED), M145-*dCas9*-*actI1* lost the ability to produce ACT on R5MS agar plates regardless of the incubation temperature (Figure 5B). M145-StrepT-switch-*dCas9*-*actI1* retained the ability to produce ACT when cultivated at 28°C. However, it lost the ability to produce ACT and retained the ability to produce RED when cultivated at 37°C (Figure 5B). The same strategy was also used for flexible control of gene expression involved in morphological differentiation. To this end, the expression of *ftsZ* was placed under the control of StrepT-switch in *S. venezuelae*. Unlike the wild-type, which formed abundant aerial hyphae and spore chains when cultivated either at 28°C or 37°C, thermal inducible repression of *ftsZ* resulted in abnormal branching hypha with almost no spore chains observed (Figure 5C). These results suggest that StrepT-switch can be used for programmable control of gene expression *via* CRISPRi based upon the thermo-inducible expression of *dCas9*.

### Induction of ZouA-dependent elevation of actinorhodin production

**Figure 6.**
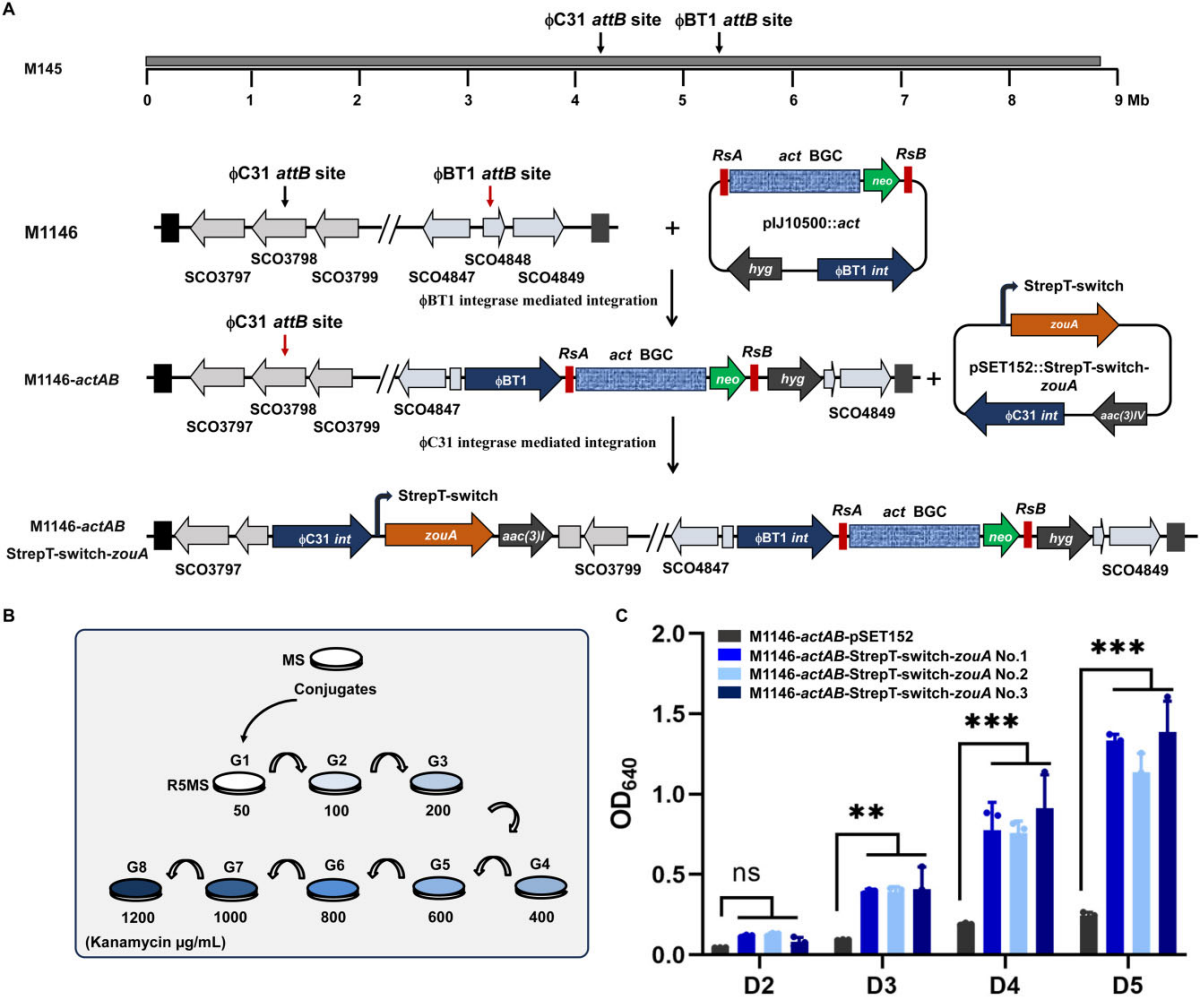
ZouA-mediated actinorhodin overproduction. (**A**) A simplified schematic diagram for the integration of the *act* gene cluster and thermal inducible *zouA* into the chromosome of *S. coelicolor* M1146. (**B**) Passage of randomly selected transformants on R5MS agar plates with increasing concentrations of kanamycin. (**C**) Actinorhodin production of recombinant strains after growing for 2–5 days in R5MS at 28°C. Error bars indicate standard deviations. Asterisks are used to denote statistical significance. Specifically, two asterisks indicate that the *P* value is <0.01, while three asterisks indicate that the *P* value is <0.001. NS: not significant.

A previous study identified a ZouA-dependent DNA amplification system in *S. kanamyceticus* (31). The ZouA amplification system consists of three genetic elements: a site-specific relaxase ZouA and two *oriT*-like recombination sites *RsA* and *RsB*. It has been successfully used for overproduction of actinorhodin in *S. coelicolor* MT1110 (31), bleomycin in *Streptomyces verticillus* (18) and spinosad in *S. coelicolor* M1146 (32). To expand the utility of StrepT-switch, the regulatory system was used to drive the expression of *zouA*. For this purpose, the *act* gene cluster was engineered with flanking sequences, including *RsA*, *RsB*, and a *neo* cassette (Figure 6A). The engineered gene cluster was transferred into *S. coelicolor* M1146 to obtain M1146-*actAB*. Incorporation of StrepT-switch-driven *zouA* generated M1146-*actAB-*StrepT-switch*-zouA*. Twelve conjugates were randomly streaked on R5MS agar plates containing 50 μg/mL kanamycin (generations one, G1), and then passaged an additional seven times (generations two–eight; G2–G8) on R5MS agar plates supplemented with increasing concentrations of kanamycin (100–1200 μg/mL) (Figure 6B). Three strains with more intense blue color were inoculated into R5MS liquid media for ACT quantification. For comparison, the empty vector pSET152 was introduced into M1146-*actAB*, and the resulting strain M1146-*actAB*-pSET152 was included to serve as a negative control. A significant increase in ACT production was observed with M1146-*actAB-*StrepT-switch*-zouA*, indicating that ZouA-dependent DNA amplification multiplies the ACT yield. Thus, two out of the three M1146-*actAB-*StrepT-switch*-zouA* strains, specifically M1146-*actAB-*StrepT-switch*-zouA* No. 1 and No. 3, were chosen for genome sequencing. Through bioinformatic analysis, it was found that no additional copy of the *act* gene cluster could be detected. However, an inversion of genomic sequences spanning ∼1.5 to 5.7 Mb was observed (Supplementary Figure S10). The inversion of genomic sequences was further confirmed by PCR-amplifications and subsequent DNA sequencing (Supplementary Figures S11 and S12). Although this inversion may contribute to the elevation of actinorhodin production, it is undoubtedly essential to conduct further investigations to delve into the mechanism underlying such genomic sequence inversion.

### The bio-switch is functional in *E. coli*

To broaden the utility of the thermal bio-switch, the intact *rheA* was placed under the control of the T7 promoter, and StrepT-switch was used to drive the expression of *neo* reporter gene in *E. coli*. Ideally, at a lower temperature, IPTG-induced RheA would repress the expression of the *neo* cassette, and the cells would be unable to grow in the presence of kanamycin. When cultivated at a higher temperature, IPTG-induced RheA would relieve its repression on the *neo* cassette, and the cells would grow well in the presence of kanamycin. Unfortunately, *E. coli* cells grew normally when cultivated either at 28°C or 37°C (Supplementary Figure S13). We speculate that this observation may arise from codon bias correlated with the expression of *rheA* in *E. coli*. To address this issue, a codon-optimized version of *rheA* was synthesized and the corresponding bio-switch was designated as EcoT-switch. When cultivated at 28°C, the cells were unable to grow in the presence of kanamycin. However, when cultivated at 37°C, they grew normally in the presence of kanamycin (Supplementary Figure S13). The results suggested that the codon-optimized version of *rheA* retains its regulatory function and maintains its ability to respond to thermal stimuli. Thus, the thermal bio-switch can be adapted for further use in *E. coli*.

## Discussion

Effective induction systems are highly sought after for diverse applications in synthetic biology and biotechnology research. Temperature is a unique input signal for the flexible control of gene expression in synthetic biotechnological applications. Temperature control is characterized by low cost, easy operability, good dispersability, and reversibility. Over the past few decades, an extensive suite of thermal induction systems has been developed for the model organism *E. coli* (33–36). Existing temperature-dependent regulators include microbial heat shock factors, membrane-associated proteins, RNA thermometers and transcriptional repressors. The most commonly used transcriptional repressors comprise a temperature-dependent transcriptional repressor TlpA from the large virulence-associated plasmid of *Salmonella typhimurium* and a temperature-sensitive variant of the bacteriophage λ repressor cI (mutant cI^857^) (36). As mentioned earlier, a temperature-inducible expression system has been developed for *Streptomyces* species (10). The system is based upon a mutated repressor TraR and its target promoter *P_tra_* from the plasmid pSN22 of *S. nigrifaciens*. It is noteworthy that the *P_tra_* promoter precedes the *traA*-*traB*-*spdB* (*tra*) operon, which is essential for the conjugative transfer of pSN22. The temperature-inducible expression system has been used for the controllable production of MDH of *Thermus flavus* in *S. lividans* (10). In this study, we report the exploration of a thermal inducible bio-switch that enables tunable and reversible control of various genes of interest. We demonstrate the versatility of this thermal bio-switch in diverse applications, such as highly efficient CRISPR/Cas9-mediated genome engineering, programmable control of antibiotic production and morphological differentiation, as well as thermal induction of ZouA for multiplication of actinorhodin production.

Upon a sudden temperature upshift, microorganisms respond by initiating the heat-shock response, leading to the prompt production of various heat-shock proteins. As mentioned earlier, the synthesis of different HSPs is under the control of four different regulatory networks in *Streptomyces* (11). For instance, the HrcA repressor is responsible for the regulation of the *groEL* genes. The HspR repressor regulates the *dnaK* operon and the *clpB* gene. A third regulator, PopR, allows tightly regulated expression of the *clpP3* and *clpP4* operon (37–40). The fourth regulator, RheA, represses the expression of divergently oriented *hsp18* gene, which encodes a small HSP protein (12). It is noteworthy that three different repressors, HrcA, HspR and RheA, specifically regulate distinct sets of heat shock genes without any cross-regulation and regulons overlap in *S. albus*. It is well established that RheA exerts its regulatory function by binding directly to its operator (*rheO*) embedded within the intergenic region between *rheA* and *hsp18*. The DNA-binding activity of the RheA repressor is modulated by high temperature to trigger its dissociation from the *rheO* operator. Circular dichroism spectroscopy revealed that RheA is capable of sensing temperature changes and undergoing conformational changes, representing a reversible transition between an active and an inactive form (12). However, the mechanism underlying the conformational change triggered by temperature remains obscure.

Due to its robustness and flexibility, CRISPR-Cas system has emerged as a versatile tool for genome engineering in many organisms. Among them, CRISPR/Cas9-based tools have been extensively used for genome editing in *Streptomyces* (41–43). However, Cas9-based editing tools have exhibited toxicity in a large number of microorganisms, including many *Streptomyces* species. These tools generally require high DNA transformation efficiency, which is unavailable for many *Streptomyces* species, thus impeding their broad applications. To address this issue, strategies mainly focus on using alternative Cas nucleases, fine-tuning Cas9 nucleases activity with anti-Cas protein and mitigating the expression of Cas9 nucleases (27). Fine-tuning the expression of Cas9 nucleases is a straightforward approach to reduce Cas toxicity while maintaining sufficient double-strand break activity for genome engineering. Since the level of Cas9 toxicity varies among different *Streptomyces* species, it is difficult to achieve optimization of Cas9 expression in a species-specific manner. In this study, the expression of Cas9 nucleases was placed under the control of the thermo-inducible bio-switch. Programmable control of gene expression can be achieved *via* CRISPR-Cas9 mediated genome engineering with easy operability of temperature control. The thermal inducible device greatly improved the DNA transformation efficiency by reducing the toxicity of Cas9 at a lower temperature, and thus greatly increased the chance to obtain CRISPR/Cas9-mediated knock-in or knock-out mutants, suggesting that the bio-switch is particularly useful for strains with low DNA transformation efficiency.

In previous studies, ZouA has been employed to achieve overproduction of actinorhodin in *S. coelicolor* MT1110 (31), bleomycin in *Streptomyces verticillus* (18), and spinosad in *S. coelicolor* M1146 (32). Earlier investigations have suggested that the relaxase plays a role in mediating the DNA amplification of gene clusters, which in turn leads to an augmentation in antibiotic yield (44,45). Two models have been proposed to account for the tandem DNA amplification phenomenon witnessed in an industrial strain of *S. kanamyceticus*. One possibility is that amplification might occur through gene duplication and subsequent amplification. Alternatively, rolling-circle replication could also be responsible. Either of these mechanisms might potentially explain the DNA amplification detected in the kanamycin-overproducing strain (44,45). However, in our current study, no evidence of DNA amplification could be discerned. Surprisingly, an inversion of genomic sequences covering ∼1.5 to 5.7 Mb was noticed. A recent study has revealed that the chromosome of *S. coelicolor* M145 contains a core region of ∼4.9 Mb in length, accompanied by two asymmetrical arms. The left arm is 1.5 Mb long, while the right arm has a length of 2.3 Mb (46). It would be highly interesting to further explore the mechanism underlying this genomic sequence inversion. Such an exploration could potentially offer valuable insights into the intricate genomic dynamics within *Streptomyces* and contribute to a more comprehensive understanding of ZouA-mediated enhancement of antibiotic production.

In summary, the thermal switch based on the temperature-sensing repressor protein enables a tunable and reversible modulation of gene expression for dynamic control of metabolic pathways in *Streptomyces*. This study has demonstrated that the thermal switch possesses attributes superior to those of commonly used inducible expression systems. Programmable control of gene expression can be achieved with the easy operability of temperature control. Moreover, the thermal bio-switch has also been adapted for use in the Gram-negative bacterium *E. coli*. Taken together, our study has expanded the toolkit of versatile induction systems that are crucial for flexible gene expression in the genus *Streptomyces*, major producers of prodigious natural products.

## Supplementary Material

gkae1236_Supplemental_File

## Data Availability

All data are incorporated into the article and its online supplementary material.
