# Supplementary material for: A tunable and reversible thermo-inducible bio-switch for streptomycetes
Source: Nucleic Acids Res. 2024 Dec 19;53(2):gkae1236. doi: 10.1093/nar/gkae1236 (PMC11754652; doi:10.1093/nar/gkae1236)
Supplement: gkae1236_Supplemental_File [file gkae1236_supplemental_file.pdf]

Supplementary Material

A tunable and reversible thermo-inducible bio-switch for streptomycetes

Lanxin Lv[1,†], Shuo Liu[1,†], Yudie Fu[1,†], Yuxin Zhang[1], Meiyan Wang[1], Jiahe Sun[2], Yi Wang[2], Yinhua Lu[3] and Guoqing Niu[1,*]

[1] College of Agronomy and Biotechnology, Southwest University, No.2, Tiansheng Road, Beibei District, Chongqing 400715, China.

[2] Integrative Science Center of Germplasm Creation in Western China (CHONGQING) Science City, Biological Science Research Center, Southwest University, No.2, Tiansheng Road, Beibei District, Chongqing, 400715, China

[3] College of Life Sciences, Shanghai Normal University, No.100 Guilin Road, Xuhui District, Shanghai, 200234, China

[†] The authors wish it to be known that, in their opinion, the first three authors should be regarded as Joint First Authors.

* Guoqing Niu

Tel: +86-23-68251883 Fax: +86-23-68251883

Email: niu062376@swu.edu.cn

Supplementary Figures

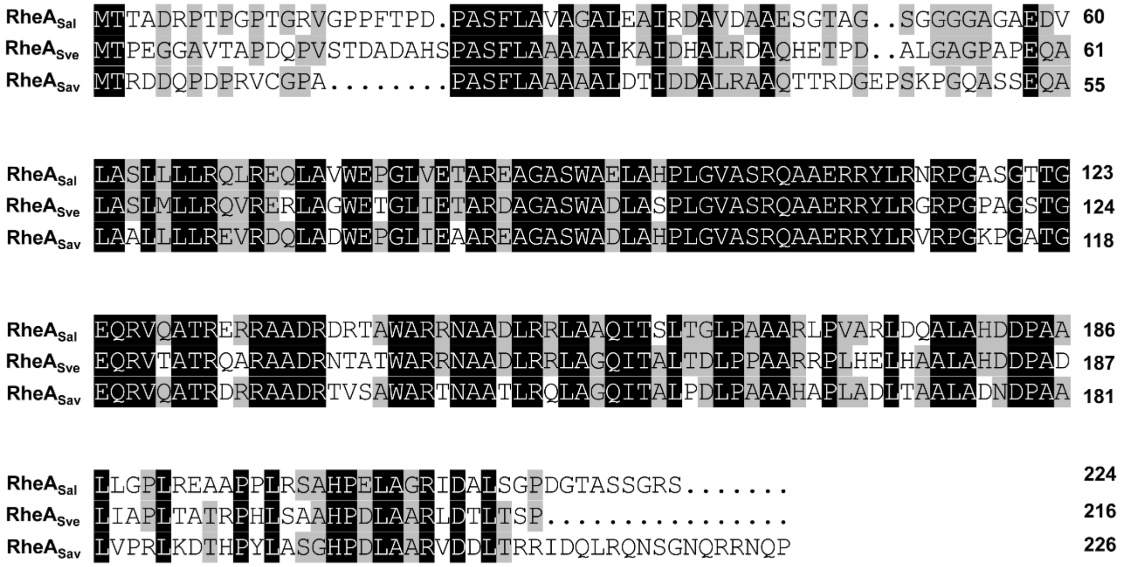

Supplementary Figure 1. **Sequence alignment of RheA in representative *Streptomyces* species.** RheA$_{Sal}$ (GenBank accession number AAC44669.1), the RheA repressor from *S. albidoflavus* J1074; RheA$_{Sve}$ (GenBank accession number CCA56456.1), the RheA orthologue from *S. venezuelae* ATCC 100712; RheA$_{Sav}$ (GenBank accession number BAC68168.1), the RheA orthologue from *S. avermitilis* MA-4680. Sequence analyses reveal that RheA$_{Sal}$ shows 65 % identity and 76 % similarity to RheA$_{Sve}$, and 62 % identity and 73 % similarity to RheA$_{Sav}$. Identical residues are shaded in black, while similar residues are shaded in gray.

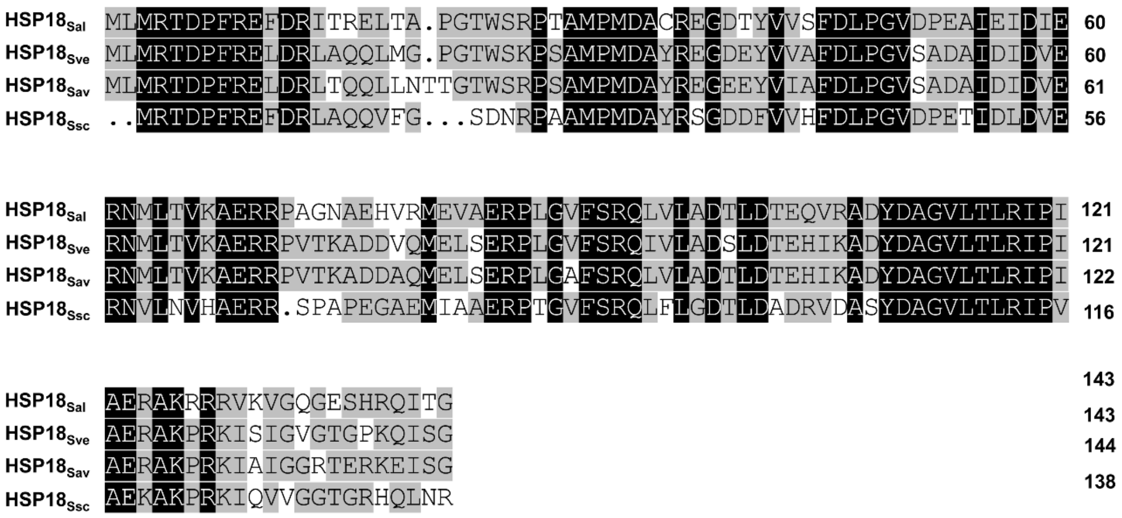

Supplementary Figure 2. **Sequence alignment of HSP18 in representative *Streptomyces* species.** $HSP18_{Sal}$ (GenBank accession number AGI86640.1), the HSP18 from *S. albidoflavus* J1074; $HSP18_{Sve}$ (GenBank accession number QER99679.1) the HSP18 orthologue from *S. venezuelae* ATCC 100712; $HSP18_{Sav}$ (GenBank accession number BAC68169.1), the HSP18 orthologue from *S. avermitilis* MA-4680; $HSP18_{Ssc}$ (GenBank accession number CBG72719.1), the HSP18 orthologue from *S. scabies* 87.22. Sequence analyses reveal that $HSP18_{Sal}$ shows 71 % identity and 87 % similarity to $HSP18_{Sve}$, 69 % identity and 84 % similarity to $HSP18_{Sav}$, and 60 % identity and 74 % similarity to $HSP18_{Ssc}$. Identical residues are shaded in black, while similar residues are shaded in gray.

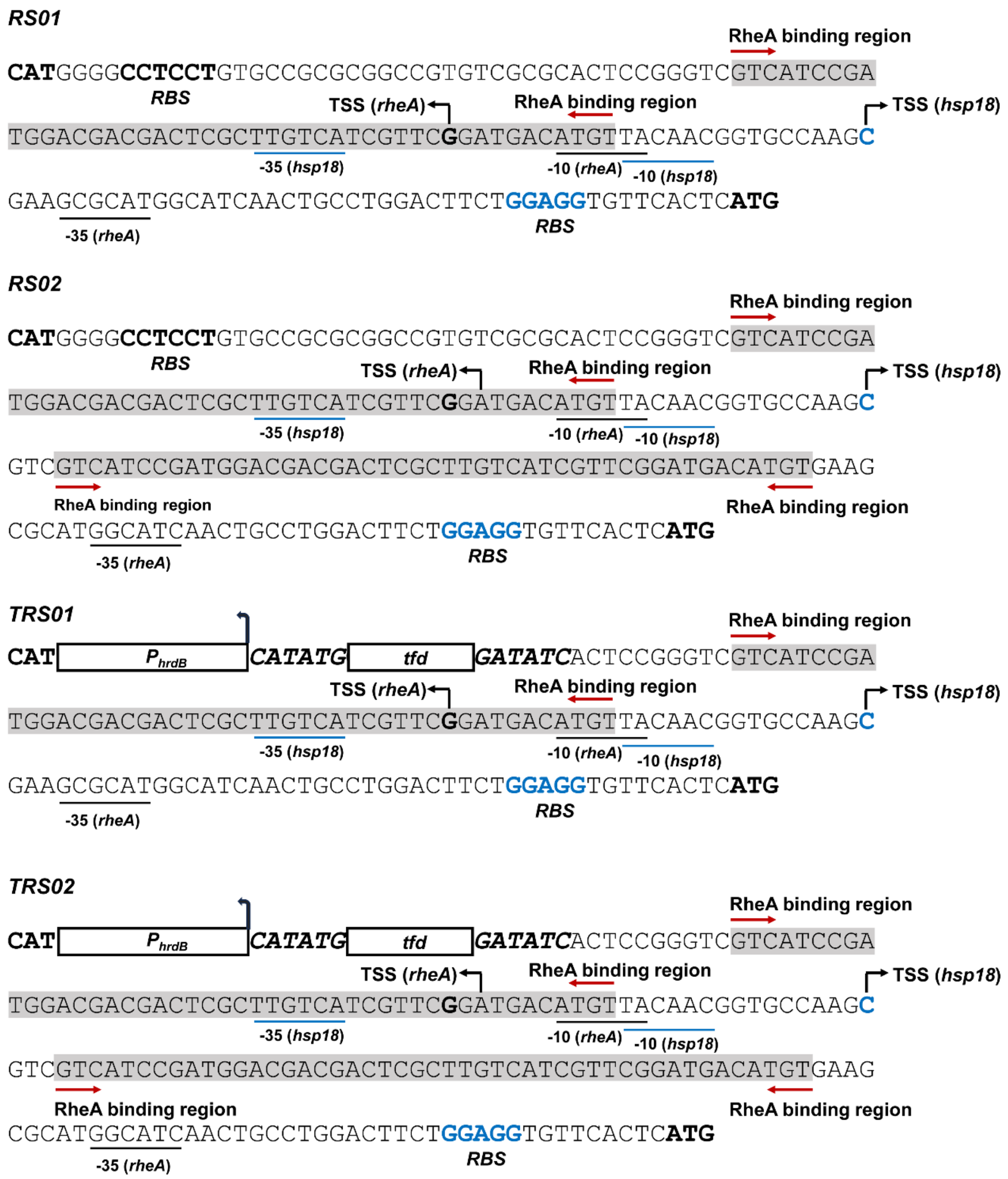

Supplementary Figure 3. **Sequences of the thermal inducible modules.** Promoter elements are as indicated. The RheA binding region is highlighted. The constitutive *hrdB* promoter is used to drive the expression of *rheA* in *TRS01* and *TRS02*. RBS, Ribosome Binding Site; TSS, Transcription Start Site; *tfd*, the *tfd* transcriptional terminator.

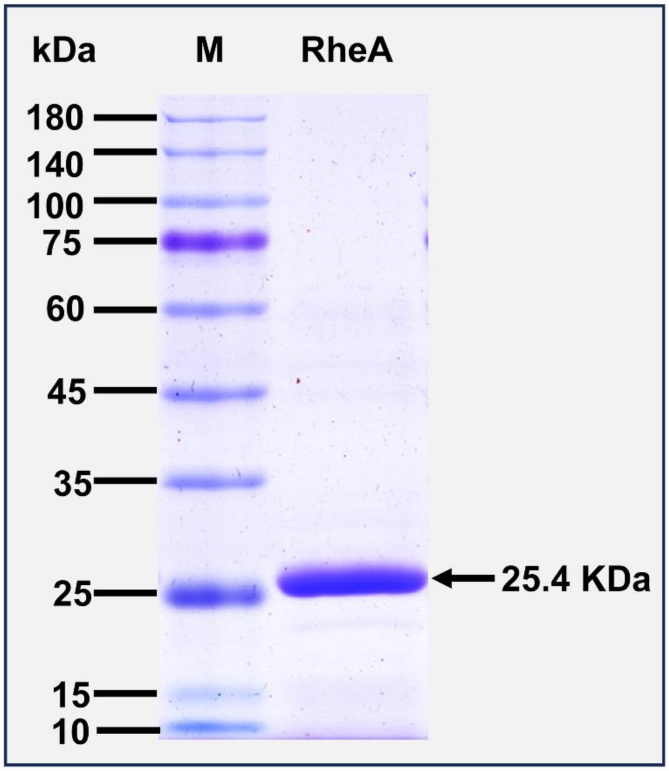

Supplementary Figure 4. SDS-PAGE analysis of purified RheA. M, molecular mass markers. Samples were separated by 12 % SDS-PAGE and stained with Coomassie brilliant blue R-250.

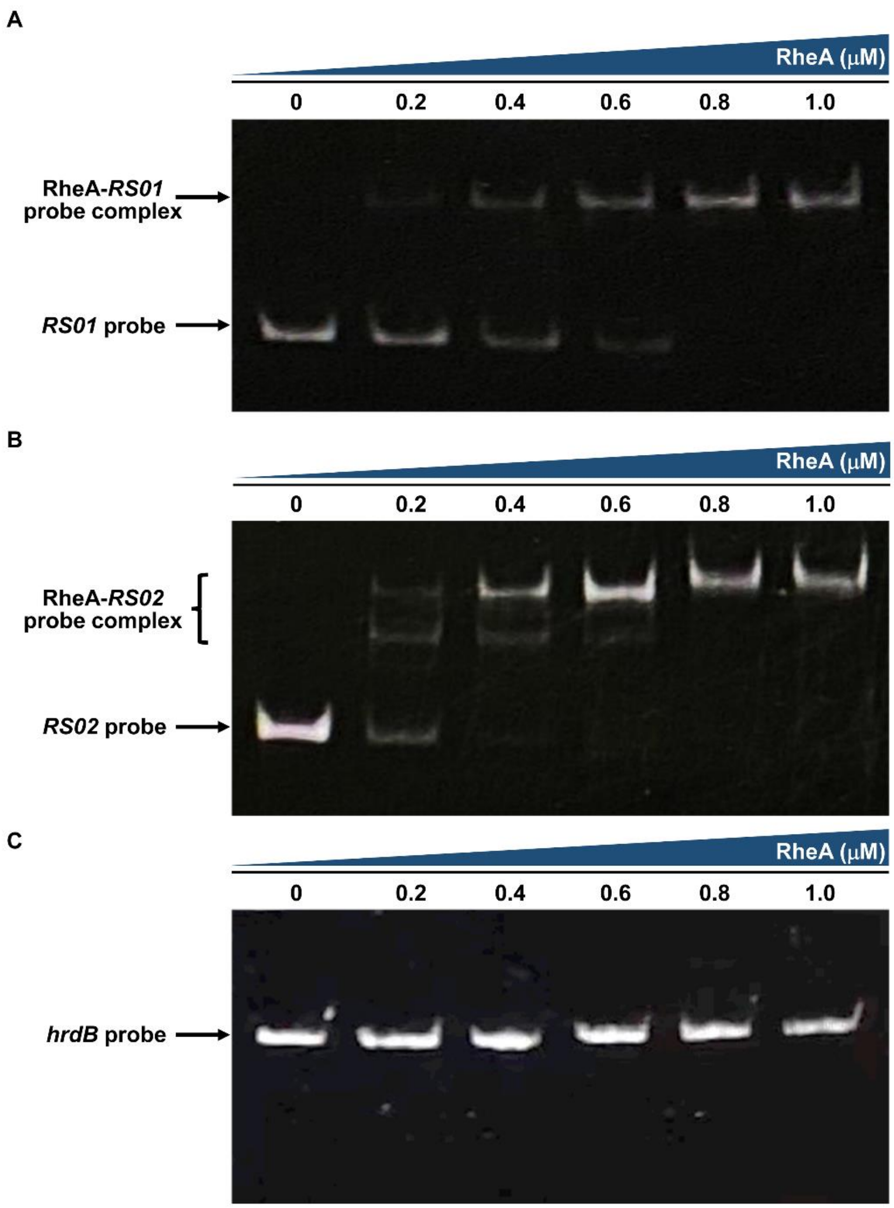

Supplementary Figure 5. Binding of purified RheA to *RS01* and *RS02* probes. Purified RheA protein was incubated with the indicated probes. The *hrdB* probe was included as a negative control. DNA-protein complexes and free probes are indicated by brackets and arrows, respectively.

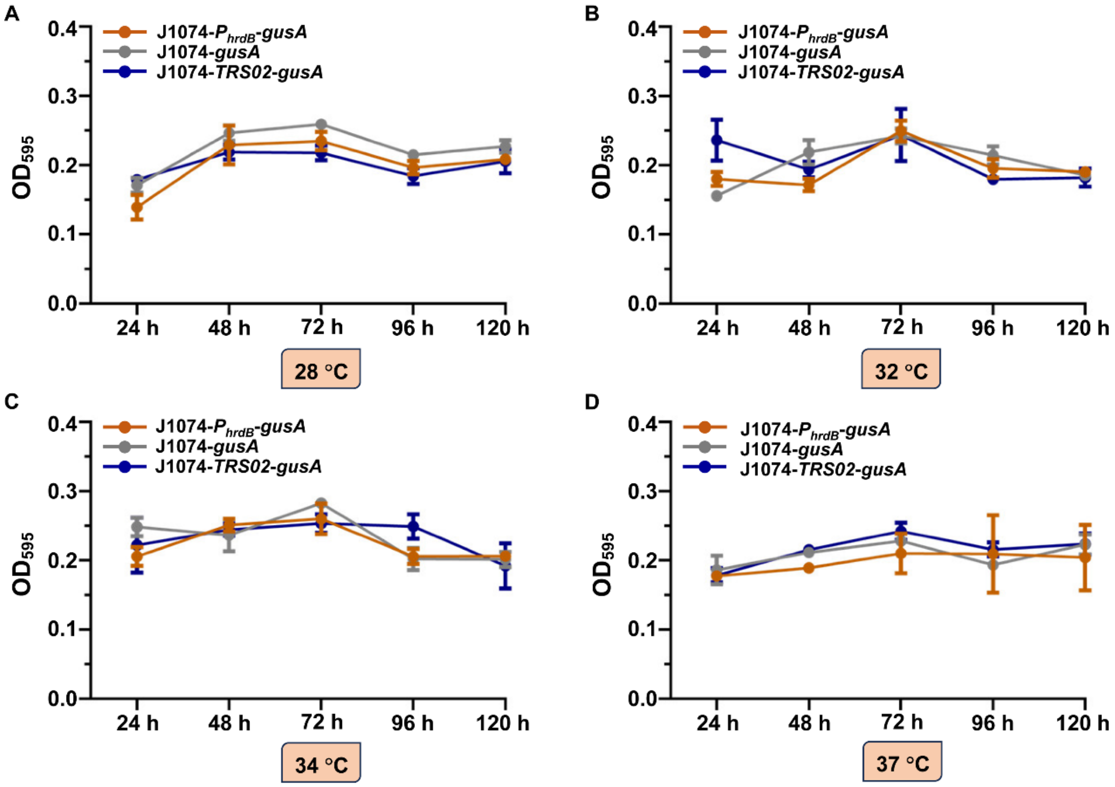

Supplementary Figure 6. Growth curves of *S. albidoflavus* J1074 derivatives. All strains were cultivated in R5A liquid media and the cultivation temperature is as indicated. Error bars represent standard deviations.

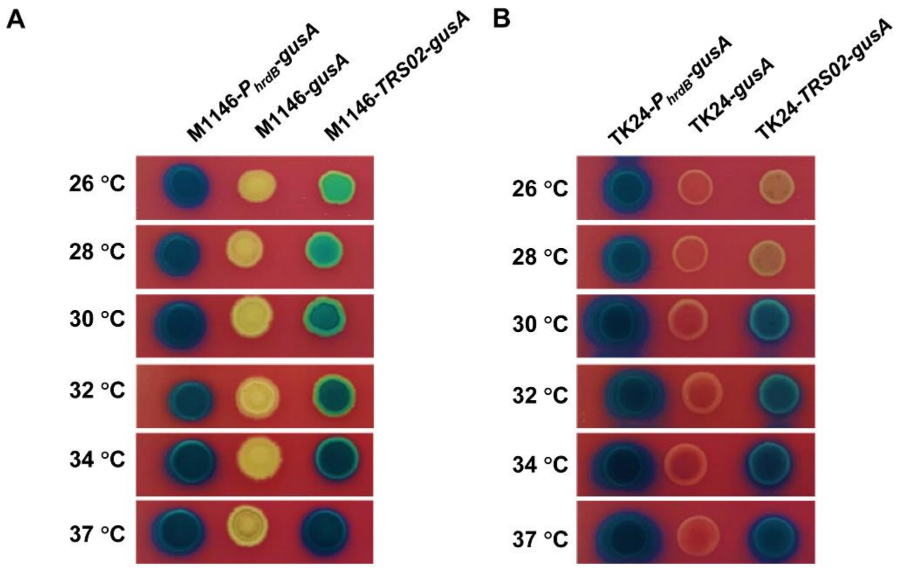

Supplementary Figure 7. GusA production in uninduced and induced states. The strains were cultivated on R2 agar plates at the indicated incubation temperature. The photograph was taken from the bottom of the plate after 5 days of cultivation. The representative image of three independent experiments with similar results is shown. M1146-P_{hrdB}-*gusA* or TK24-P_{hrdB}-*gusA* was included to serve as a positive control, while M1146-*gusA* or TK24-*gusA* was used as a negative control.

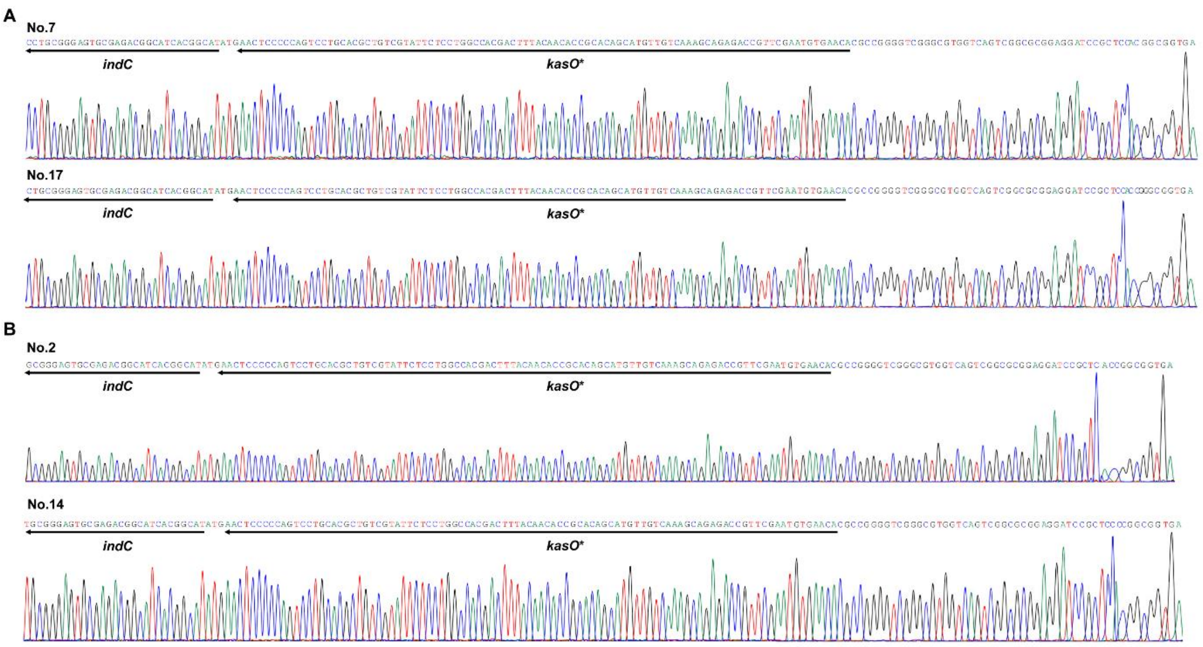

Supplementary Figure 8. Verification of CRISPR/Cas9-mediated target knock-in by DNA sequencing. (A) Sequencing the PCR amplicons obtained with the genomic DNA from KI *tipA*-Cas9 as the template. (B) Sequencing the PCR amplicons acquired using the genomic DNA from KI StrepT-switch-Cas9 as the template. Five PCR amplicons from KI *tipA*-Cas9 or KI StrepT-switch-Cas9 have been confirmed through DNA sequencing. Two examples for each are presented here, and the numbering is consistent with that in Figure 4 of the main text.

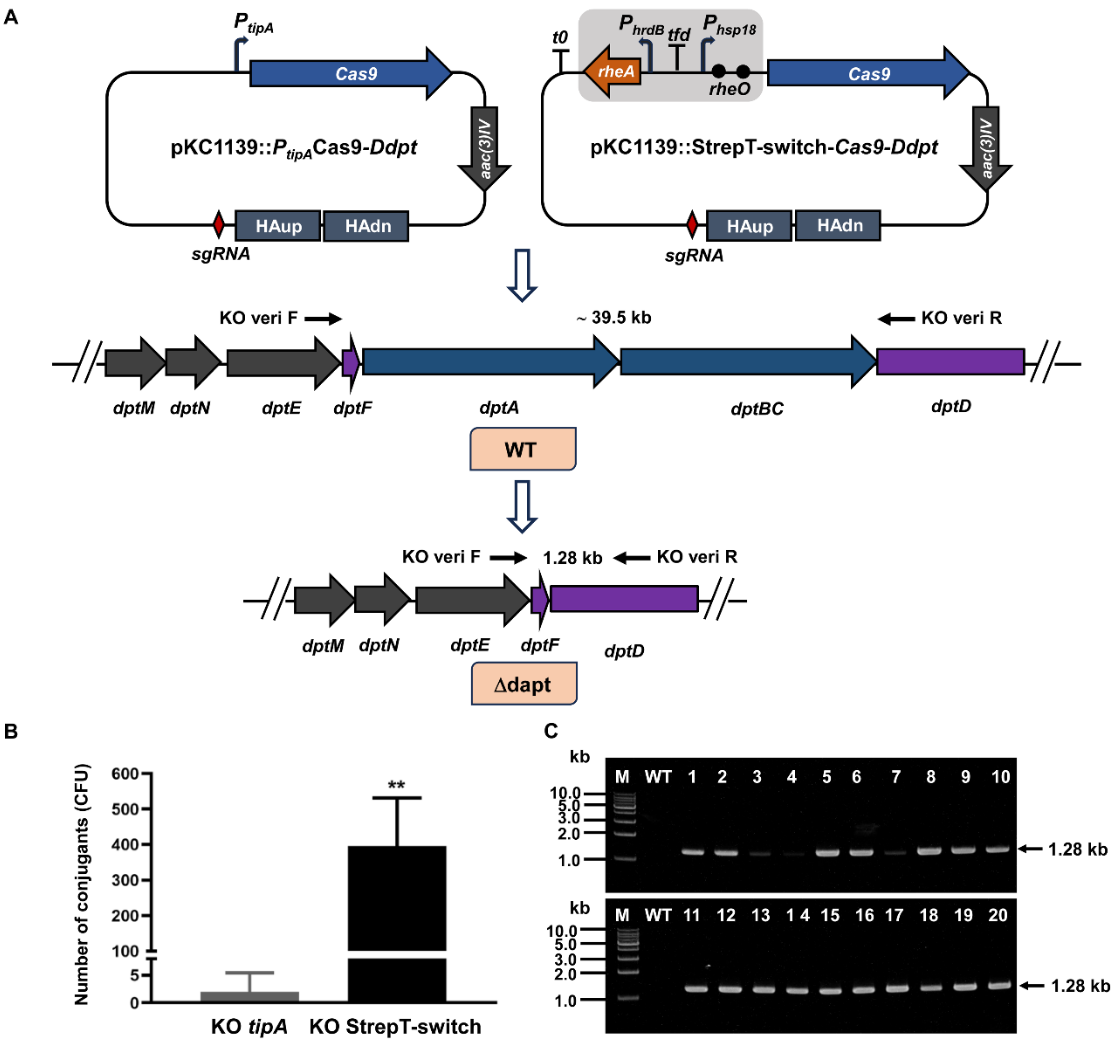

Supplementary Figure 9. Thermal inducible CRISPR/Cas9-mediated target knock-out. (A) CRISPR/Cas9-mediated knock-out of approximate 39.5 kb of the daptomycin gene cluster from the chromosome of *S. roseosporus*. The *tipA* promoter or StrepT-switch was employed to drive the expression of Cas9. (B) Comparison of transformation efficiency between KO *tipA* and KO StrepT-switch. (C) Verification of transformants by PCR amplifications. The expected size of PCR amplicons is as indicated. "kb" represents kilobase, and "M" denotes DNA Ladder. Twenty transformants (1-20) were randomly selected for DNA extractions and subsequent PCR amplifications. The templated from *Streptomyces roseosporus* (WT) was included to serve as a negative control.

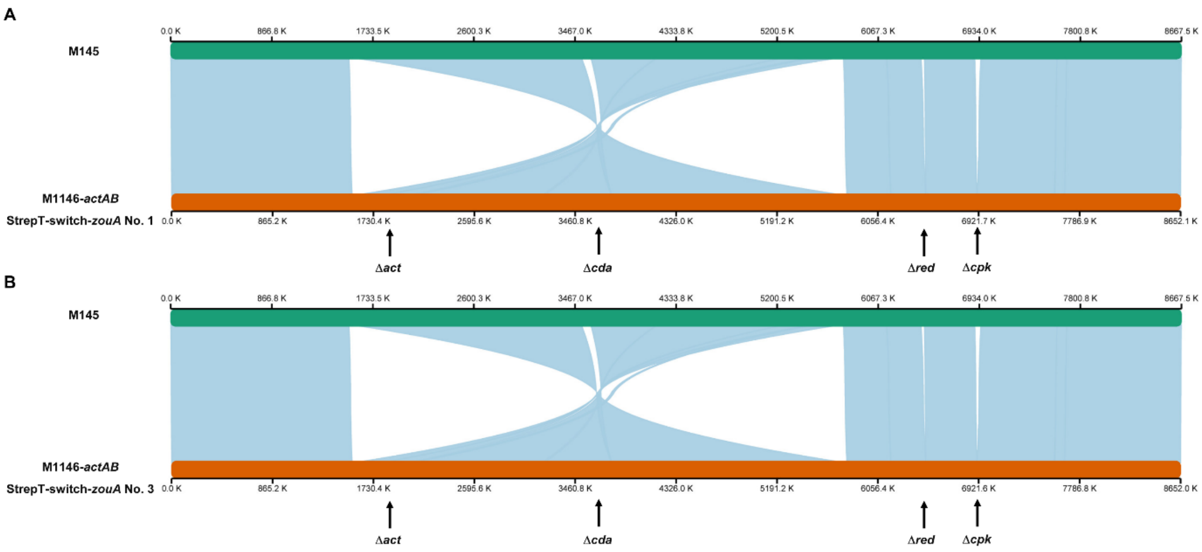

Supplementary Figure 10. Genome synteny analysis of the sequenced genomes. (A) Comparative analysis between *S. coelicolor* M145 (M145) and M1146-*actAB*-StrepT-switch-*zouA* No.1. (B) Comparative analysis between *S. coelicolor* M145 (M145) and M1146-*actAB*-StrepT-switch-*zouA* No.3. The positions of the deletion of gene clusters for actinorhodin (*act*), undecylprodigiosin (*red*), calcium dependent antibiotic (*cda*) and coelimycin P1 (*cpk*) were indicated as shown in the figure.

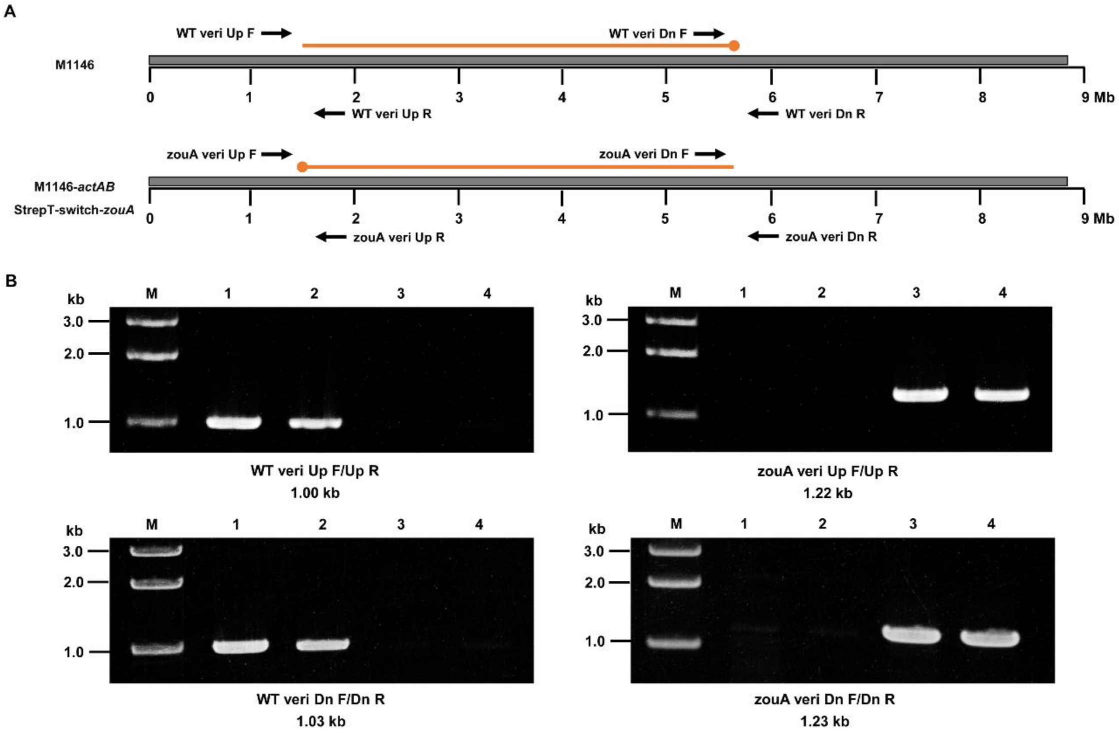

Supplementary Figure 11. Confirmation of genomic sequence inversion by PCR amplifications. (A) A simplified schematic illustration showing the inversion of genomic sequences that span approximately 1.5 M to 5.7 M. The positions of primers used for PCR amplifications are indicated as shown. (B) Verification of genomic sequence inversion by PCR amplifications. The anticipated size of PCR amplicons is as indicated. “kb” represents kilobase, and “M” denotes DNA Ladder. PCR templates include genomic DNAs from the following strains: *S. coelicolor* M1146 (1), M1146-*actAB*-pSET152 (2), M1146-*actAB*-StrepT-switch-*zouA* No. 1 (3), and M1146-*actAB*-StrepT-switch-*zouA* No. 3 (4).

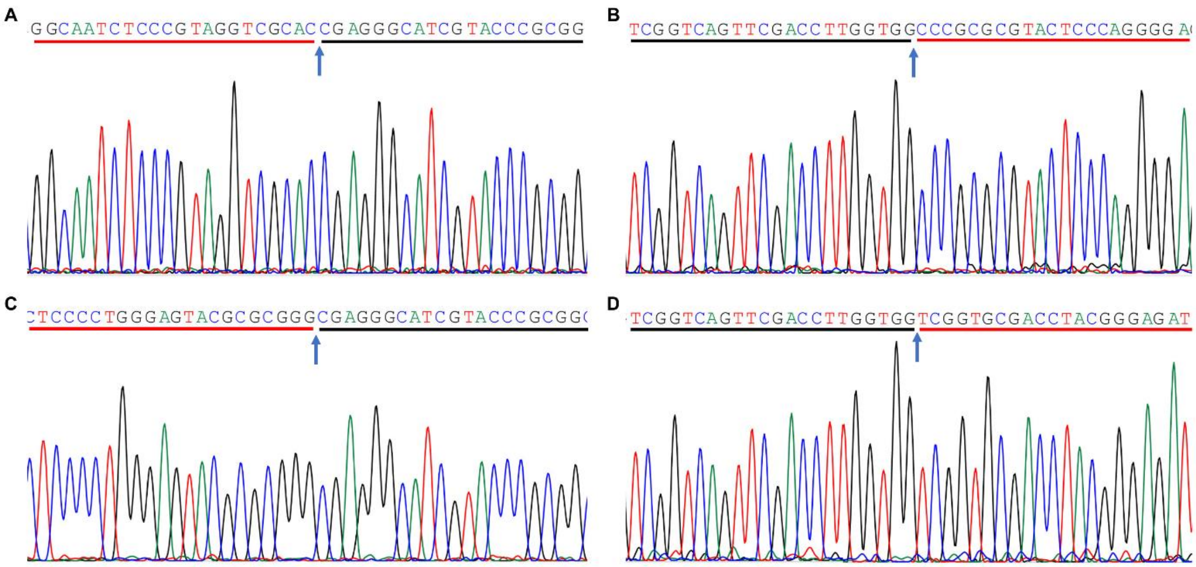

Supplementary Figure 12. Confirmation of genomic sequence inversion by DNA sequencing. (A) Sequencing the PCR amplicon acquired using the genomic DNA from *S. coelicolor* M1146 as the template with primer pair WT veri Up F/Up R. (B) Sequencing the PCR amplicon acquired using the genomic DNA from *S. coelicolor* M1146 as the template with primer pair WT veri Dn F/Dn R. (C) Sequencing the PCR amplicon generated with the genomic DNA from M1146-*actAB*-StrepT-switch-*zouA* No. 1 as the template with primer pair zouA veri Up F/Up R. (D) Sequencing the PCR amplicon generated with the genomic DNA from M1146-*actAB*-StrepT-switch-*zouA* No. 1 as the template with primer pair zouA veri Dn F/Dn R. Of note is that sequences of PCR amplicons with M1146-*actAB*-StrepT-switch-*zouA* No. 3 as the template are precisely identical to those of PCR amplicons with M1146-*actAB*-StrepT-switch-*zouA* No. 1 as the template.

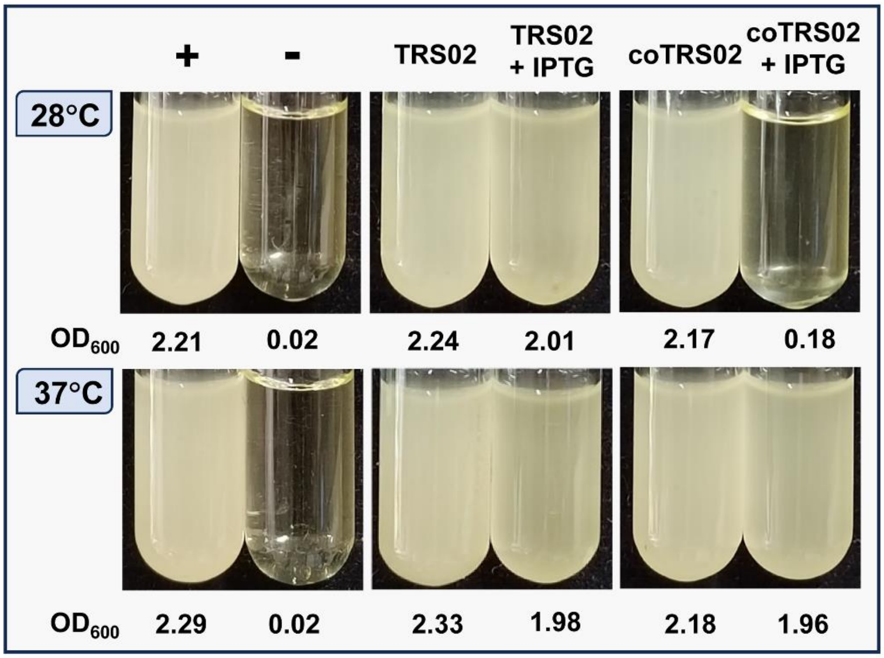

Supplementary Figure 13. Evaluation of the thermal bio-switch in *E. coli*. The StrepT-switch fails to switch off the expression of the *neo* cassette when the strain is cultivated at 28 °C. After codon optimization, the EcoT-switch is functional in *E. coli*. When cultivated at 28 °C, RheA repressor switches off expression of the *neo* cassette, and cells are unable to grow in the presence of kanamycin. When cultivated at 37 °C, the thermo-sensing repressor relieves its repression on the *neo* cassette, and cells grow normally in the presence of kanamycin.

Table S1. Bacterial strains used in this study.

Bacteria strains	Relevant Characteristics	Reference/Source
***Streptomyces* Strains**		
S. albidoflavus J1074 (J1074)	A derivative of *S. albus* G1 defective in the *Sal*IG1 restriction-modification system	(1)
J1074-P_{hrdB}-*neo*	*S. albidoflavus* J1074 containing pSET152::P_{hrdB}-*neo*, Apr^R	(2)
J1074-*neo*	*S. albidoflavus* J1074 containing pSET152::*neo*, Apr^R	(2)
J1074-*RS01-neo*	*S. albidoflavus* J1074 containing pSET152:: *RS01-neo*, Apr^R, Kan^R	This work
J1074-*TRS01-neo*	*S. albidoflavus* J1074 containing pSET152:: *TRS01-neo*, Apr^R, Kan^R	This work
J1074-*TRS02-neo*	*S. albidoflavus* J1074 containing pSET152:: *TRS02-neo*, Apr^R, Kan^R	This work
J1074-P_{hrdB}-*gusA*	*S. albidoflavus* J1074 containing pSET152:: P_{hrdB}-*gusA*, Apr^R	This work
J1074-*gusA*	*S. albidoflavus* J1074 containing pSET152:: *gusA*, Apr^R	This work
J1074-*TRS02-gusA*	*S. albidoflavus* J1074 containing pSET152:: *TRS02-gusA*, Apr^R	This work
J1074-P_{hrdB}-*egfp*	*S. albidoflavus* J1074 containing pSET152:: P_{hrdB}-*egfp*, Apr^R	This work
J1074-*egfp*	*S. albidoflavus* J1074 containing pSET152:: *egfp*, Apr^R	This work
J1074-*TRS02-egfp*	*S. albidoflavus* J1074 containing pSET152:: *TRS02-egfp*, Apr^R	This work

KI *tipA*	*S. albidoflavus* J1074 containing pKCCas9::*kasO**-KI *tipA*, Apr^R	This work
KI StrepT-switch	*S. albidoflavus* J1074 containing pKCCas9:: *kasO**-KI StrepT-switch, Apr^R	This work
S. venezuelae ATCC 10712 (Svn, WT)	Wild-type, chloramphenicol and jadomycin B producer	ATCC
S*vn* StrepT-switch-*dCas9-ftsZ*	*S. venezuelae* ATCC 10712 containing pSET152::StrepT-switch-*dCas9-ftsZ*, Apr^R	This work
S. coelicolor M145 (M145)	A derivative of the wild-type strain A3(2) lacking plasmids SCP1 and SCP2	(3)
M145-*dCas9-actI1*	*S. coelicolor* M145 containing pSET152::*dCas9-actI1*, Apr^R	(2)
M145 StrepT-switch-*dCas9-actI1*	*S. coelicolor* M145 containing pSET152::StrepT-switch-*dCas9-actI1*, Apr^R	This work
S. coelicolor M1146 (M1146)	An engineered derivative of *S. coelicolor* M145 lacking gene clusters for actinorhodin (*act*), undecylprodigiosin (*red*), calcium dependent antibiotic (*cda*) and coelimycin P1 (*cpk*)	(4)
M1146-*actAB*	*S. coelicolor* M1146 containing pIJ10500::*act*, Hyg^R	This work
M1146-*actAB*-pSET152	A derivative of M1146-*act* containing the empty pSET152, Apr^R	This work
M1146-*actAB*-StrepT-switch-*zouA*	A derivative of M1146-*act* containing pSET152::StrepT-switch-*zouA*, Apr^R	This work
M1146-P_{hrdB}-*gusA*	*S. coelicolor* M1146 containing pSET152:: P_{hrdB}-*gusA*, Apr^R	This work
M1146-*gusA*	*S. coelicolor* M1146 containing pSET152::*gusA*, Apr^R	This work

M1146-*TRS02-gusA*	*S. coelicolor* M1146 containing pSET152::*TRS02-gusA*, Apr^R	This work
S. lividans TK24	A plasmid-free derivative strain of *S. lividans* 66	(5)
TK24-P_{hrdB}-*gusA*	*S. lividans* TK24 containing pSET152::P_{hrdB} -*gusA*, Apr^R	This work
TK24-*gusA*	*S. lividans* TK24 containing pSET152::*gusA*, Apr^R	This work
TK24-*TRS02-gusA*	*S. lividans* TK24 containing pSET152::*TRS02-gusA*, Apr^R	This work
S. roseosporus NRRL 15998	Wild-type, daptomycin producer	(6)
KO *tipA*	*S. roseosporus* NRRL 15998 containing pKCCas9::*dptABC* KO *tipA*, Apr^R	This work
KO StrepT-switch	*S. roseosporus* NRRL 15998 containing pKCCas9::*dptABC* KO StrepT-switch, Apr^R	This work
***E. coli* Strains**		
DH5α	F$^-$ φ80*lac*ZΔM15 Δ(*lac*ZYA-*arg*F)U169 *rec*A1 *end*A1 *hsd*R17(r_K^-, m_K^+) *pho*A *sup*E44 λ$^-$ *thi*-1 *gyr*A96 *rel*A1	ThermoFisher Scientific
JM109	*endA*1 *recA*1 *gyrA*96 *thi*-1 *hsdR*17(rk-, mk+) *relA*1 *supE*44 D (*lac-proAB*) [*F' traD36 proAB laqI*^{q}*ZΔM*15]	ThermoFisher Scientific
ET12567(pUZ8002)	*dam dcm hsdS cat tet/*pUZ8002	(7)
BW25113	K-12 derivative; Δ*araBAD* Δ*rhaBAD*	(8)
BL21 (DE3)	*fhuA2* [*lon*] *ompT gal* (*λ DE3*) [*dcm*] Δ*hsdS*, *λ DE3* = *λ sBam*HIo Δ*Eco*RI-B *int*::(*lac*I::P*lacUV5*::*T7 gene1*) *i21* Δ*nin5*	New England Biolabs
BL21-P_{neo}-*neo*	*E. coli* BL21 (DE3) containing pET23b::P_{neo}-*neo*, Amp^R, Kan^R	This work
BL21-*neo*	*E. coli* BL21 (DE3) containing pET23b::*neo*, Amp^R	This work

BL21-StrepT-switch	*E. coli* BL21 (DE3) containing pET23b::StrepT-switch-*neo*, Amp^{R}, Kan^{R}	This work
BL21-EcoT-switch	*E. coli* BL21 (DE3) containing pET23b::EcoT-switch-*neo*, Amp^{R}, Kan^{R}	This work

Apr^{R}, apramycin resistance; Hyg^{R}, hygromycin resistance; Amp^{R}, ampicillin resistance; Kan^{R}, kanamycin resistance.

Table S2. Plasmids used in this study.

Plasmids	Relevant Characteristics	Reference/Source
pIJ790	λ-RED (*gam, bet, exo*), *cat*, *araC*, *rep101*^{ts}	(9)
pUC119::*neo*	pUC119 containing kanamycin resistance gene (*neo*), Kan^R	(10)
pIJ8660	An integrative vector containing the reporter gene *egfp* between the major transcription terminator of phage *tfd* and *t0*, Apr^R	(11)
pSET152	Integrative vector, Apr^R	(12)
pSET152::P_{hrdB}-*neo*	pSET152 containing *neo* driven by *hrdB* promoter, Apr^R, Kan^R	(13)
pSET152::*neo*	pSET152 containing the promoter-less *neo*, Apr^R	(2)
pSET152::*RS01*-neo	pSET152 containing *rheA* driven by its native promoter and the *neo* cassette driven by the *hsp18* promoter, Apr^R, Kan^R	This work
pSET152::*TRS01-neo*	pSET152 containing *rheA* driven by the constitutive *hrdB* promoter and the *neo* cassette driven by the *hsp18* promoter, Apr^R, Kan^R	This work
pSET152::*TRS02-neo*	pSET152 containing *rheA* driven by the constitutive *hrdB* promoter and the *neo* cassette driven by a modified *hsp18* promoter with the insertion of an additional copy of *rheO*, Apr^R, Kan^R	This work
pSET152::P_{hrdB}-*gusA*	pSET152 containing *gusA* driven by the *hrdB* promoter, Apr^R	(14)
pSET152::*gusA*	pSET152 containing the promoter-less *gusA*, Apr^R	(14)
pSET152::*TRS02-gusA*	A derivative of pSET152::*TRS02-neo* with the replacement of *neo* by *gusA*, Apr^R	This work

pSET152::P_{hrdB}-*egfp*	pSET152 containing *egfp* driven by the *hrdB* promoter, Apr^R	This work
pSET152::*egfp*	pSET152 containing the promoter-less *egfp*, Apr^R	This work
pSET152::*TRS02-egfp*	A derivative of pSET152::*TRS02-neo* with the replacement of *neo* by *egfp*, Apr^R	This work
pSET152::StrepT-switch-*zouA*	A derivative of pSET152 containing *zouA* driven by StrepT-switch, Apr^R	This work
pSET152::*dCas9-actI1*	A derivative of pSET152 containing *dCas9* driven by the *ermE** promoter with sgRNA targeting *actI-ORF1*, Apr^R	(15)
pSET152::StrepT-switch-*dCas9-actI1*	A derivative of pSET152 containing *dCas9* driven by StrepT-switch with sgRNA targeting *actI-ORF1*, Apr^R	This work
pSET152::StrepT-switch-*dCas9-ftsZ*	A derivative of pSET152 containing *dCas9* driven by StrepT-switch with sgRNA targeting *ftsz*, Apr^R	This work
pKCcas9dO	*acc(3)IV*, pSG5, *tipA-Scocas9*, Apr^R	(16)
pKCcas9::*kasO**-KI tipA	A derivative of pKC1139 containing *tipA*-driven *Cas9* with sgRNA targeting *SSHG_00313* (*indC*), and the *kasO** promoter situated between homologous arms upstream and downstream of *indC*, Apr^R	This work
pKCcas9::*kasO**-KI StrepT-switch	A derivative of pKC1139 containing StrepT-switch-driven *Cas9* with sgRNA targeting *SSHG_00313* (*indC*), and the *kasO** promoter situated between homologous arms upstream and downstream of *indC*, Apr^R	This work
pKCcas9::d*dptABC*	A derivative of pKC1139 containing *Cas9* driven by the *tipA* promoter, *dpt* guide-RNA and	This work

	homologous region flanking genes *dptA* and *dptBC*, Apr^R	
pKCcas9::StrepT-switch-d*dptABC*	A derivative of pKC1139 containing *Cas9* driven by StrepT-switch, *dpt* guide-RNA and homologous region flanking genes *dptA* and *dptBC*, Apr^R	This work
pIJ10500	A derivative of pMS82 containing φBT1 integrase gene and hygromycin resistance cassette, Hyg^R	(17)
pIJ10500::*act*	A derivative of pIJ10500 containing the *act* gene cluster with flanking *RsA* and *RsB* sites and the *neo* cassette, Hyg^R	This work
pET23b	Expression vector, Amp^R	Novagen
pET23b::P_{neo}-*neo*	pET23b containing the *neo* cassette and its promoter, Amp^R, Kan^R	This work
pET23b::*neo*	pSET152 containing the promoter-less *neo* cassette, Amp^R	This work
pET23b::StrepT-switch-*neo*	pET23b containing the coding region of *rheA* driven by T7 promoter, and the *neo* cassette driven by *RS02*, Amp^R, Kan^R	This work
pET23b::EcoT-switch-*neo*	pET23b containing the coding region of *rheA* driven by T7 promoter, and the *neo* cassette driven by a modified *lacUV5* promoter with insertion of two *rheO* sites, Amp^R, Kan^R	This work
pET28a	Expression vector, Kan^R	Novagen
pET28a::*rheA*	pET28a containing the coding region of *rheA* driven by T7 promoter, Kan^R	This work

Apr^R, apramycin resistance; Hyg^R, hygromycin resistance; Amp^R, ampicillin resistance; Kan^R, kanamycin resistance.

Table S3. Primers used in this study.

**Primers**	**Sequence (5´-3´)** [a,b]	**Purpose**
RS01 F	aattTCTAGAGCCCGAGCACCAGTCGCGTAC	Construction of pSET152::*RS01-neo*
RS01 R	GAGTGAACACCTCCAGAAGTCC	Construction of pSET152::*RS01-neo*
neo orfF	ATGATTGAACAAGATGGATTGC	Construction of pSET152::*RS01*-neo
neo orfR	aattGAATTCGAGCTCGGTACCCGAACCCCAG	Construction of pSET152::*RS01*-neo
t0 F	aattTCTAGAATGCATTCAGCAGGTGGAAGAGGGAC	Construction of pSET152::*RS01-neo*
t0 R	*CTGGTGCTCGGGCGGATCC*GTCCAGTAATGACCTCAGAACTCC	Construction of pSET152::*RS01-neo*
hrdBp F	*GGGCGGTCGGCGGTGGTCAT*GAACAACCTCTCGGAACGTTG	Construction of pSET152::*TRS01-neo* and pSET152::*TRS02-neo*
hrdBp R	*GACGACAAAACTTTCATATG*CCGCCTTCCGCCGGAACG	Construction of pSET152::*TRS01-neo* and pSET152::*TRS02-neo*
tfd F	CATATGAAAGTTTTGTCGTCTTTCCAGACG	Construction of pSET152::*TRS01-neo* and pSET152::*TRS02-neo*
tfd R	*CGGATGACGACCCGGAGTGATATC*CCCGGGAACCCGGCCGCG	Construction of pSET152::*TRS01-neo* and pSET152::*TRS02-neo*

gusA F	*GACTTCTGGAGGTGTTCACTC*ATGACCGGTCTG CGGCCC	Construction of pSET152::*TRS02-gusA*
gusA R	*CAGCTATGACATGATTACGAATTC*TCACTGCTTC CCGCCCTG	Construction of pSET152::*TRS02-gusA*
egfp F	*TTCTGGAGGTGTTCACTC*CATATGGTGAGCAAG GGCGAGGAG	Construction of pSET152::*TRS02-egfp*
egfp R	*CAGCTATGACATGATTAC*GAATTCTTACTTGTA CAGCTCGTCCATGC	Construction of pSET152::*TRS02-egfp*
indC sgRNA F	aattACTAGTCCGGGGGAAGAGCTGCCCCCGTT TTAGAGCTAGAAATAG	Construction of pKCCas9::*kasO**-KI StrepT-switch
indC sgRNA R	CTCAAAAAAAGCACCGACTC	Construction of pKCCas9::*kasO**-KI StrepT-switch
indC Up F	*GTCGGTGCTTTTTTTGAG*TCGCTGCTCGGCTTC GCGAG	Construction of pKCCas9::*kasO**-KI StrepT-switch
indC Up R	CGCCGGGGTCGGGCGTGG	Construction of pKCCas9::*kasO**-KI StrepT-switch
kasO* F	*CCACGCCCGACCCCGGCG*TGTTCACATTCGAA CGGTCTCTG	Construction of pKCCas9::*kasO**-KI StrepT-switch
kasO* R	AACTCCCCCAGTCCTGCACG	Construction of pKCCas9::*kasO**-KI StrepT-switch
indC Dn F	*AGCGTGCAGGACTGGGGGAGTT*ATGCCGTGAT GCCGTCT	Construction of

		pKCCas9::*kasO**-KI StrepT-switch
indC Dn R	aattAAGCTTCCGGGTCAGTGTCGAGCAG	Construction of pKCCas9::*kasO**-KI StrepT-switch
KI veri F	TGTTCACATTCGAACGGTCT	Verification of *kasO** promoter knock-in
KI veri R	CCGCCGATGAACAGTTCCCC	Verification of *kasO** promoter knock-in
ftsZ sgRNA F	aattACTAGTAGACCATGTCGGCCCCCTTGGTTTTAGAGCTAGAAATAGCAAG	Construction of pSET152::StrepT-switch-dCas9-*ftsZ*
ftsZ sgRNA R	aattGAATTCGGGTGTACATCCAGTAATG	Construction of pSET152::StrepT-switch-dCas9-*ftsZ*
rheA orfF	aattCATATGATGACCACCGCCGACCGCCC	Construction of pET28a::*rheA*
rheA orfR	aattGAATTCTCAGGACCGCCCGGACGAGG	Construction of pET28a::*rheA*
rheO F	GATATCACTCCGGGTCGTCATCCG	PCR amplification of RS01 and RS02 probes
rheO R	GAGTGAACACCTCCAGAAGT	PCR amplification of RS01 and RS02 probes
dpt sgRNA F	aattACTAGTTCTCCTCGACTACCTTCGACGTTTTAGAGCTAGAAATAG	Knock-out of *daptABC*
dpt Up F	*GGCACCGAGTCGGTGCTTTTTTTGAG*GTCGGCCACTTCCGCGCC	Knock-out of *daptABC*
dpt Up R	CGGTCGGCCAACTGGGGC	Knock-out of *daptABC*

dpt Dn F	*GCCCCAGTTGGCCGACCG*CGCAGCGCGCGATGGAGGAC	Knock-out of *daptABC*
dpt Dn R	aattAAGCTTAGCCGCTGGATGACCGCC	Knock-out of *daptABC*
KO veri F	GCCGGTGACGCACCCCTG	Verification of knock-out mutants
KO veri R	GTCGGCGACCCGGGGTTC	Verification of knock-out mutants
RsA F	aatTCTAGACGGCCCGCTCAACCCCCGCT	Construction of pIJ10500::*act*
RsA R	aattAAGCTTAATTAACCCGTGACCGACACCCGCTGT	Construction of pIJ10500::*act*
RsB F	aattGATATCGCGATGAAGCTCGTCGAGATC	Construction of pIJ10500::*act*
RsB R	aattAAGCTTAGATCTGATGCCGAACGCGTGGTTCAG	Construction of pIJ10500::*act*
ZouA F	aattGAATTCAGGGAGATTGGCCCGGTGCAC	Construction of pSET152::StrepT-switch-*zouA*
ZouA R	aattCATATGGGATGGGTGACCATGATCG	Construction of pSET152::StrepT-switch-*zouA*
WT veri Up F	GTGCTGCACCTCGACGACATC	Verification of the inversion of genomic sequences
WT veri Up R	GTAGGCCCCCATGGTGTAGGC	Verification of the inversion of genomic sequences
zouA veri Up F	CTGGCCGGACTTCTGTGGATGG	Verification of the inversion of genomic sequences

zouA veri Up R	CGACGCAGTGCATGTTCCACAC	Verification of the inversion of genomic sequences
WT veri Dn F	ATGCGGAAGATACTCGTCGTCG	Verification of the inversion of genomic sequences
WT veri Dn R	CGCATCCAGGTCTCGTCGAAC	Verification of the inversion of genomic sequences
zouA veri Dn F	CATCAGGGCCGCCGAAAGGAGC	Verification of the inversion of genomic sequences
zouA veri Dn R	GACGAACGGGTCCGACGAGGGG	Verification of the inversion of genomic sequences

[a] Underlined sequences for restriction enzyme recognition sites; [b] italicized sequences for overlapping between DNA sequences.

Table S4. Sequences of sgRNA and homologous fragments used for CRISPR/Cas9-mediated genome editing.

Nucleotide sequences
**sgRNA and donor sequences of pKCcas9::*kasO**-KI StrepT-switch**
sgRNA sequences are highlighted in yellow, and donor sequences are in bold. The sequences of *kasO**are in orange, and the restriction sites are underlined.
(*Spe*I)<u>ACTAGT</u>CCGGGGGAAGAGCTGCCCCCGTTTTAGAGCTAGAAATAGCAAGTTAAAATAAGGCTAGTCCG<br>TTATCAACTTGAAAAAGTGGCACCGAGTCGGTGCTTTTTTTGAG**TCGCTGCTCGGCTTCGCGAGCCCGCTCG**<br>**CCGCCACCGTCCTCGGCTACCTCTTCCTGCGACAGGGCCTGACGCTCGTCCAGGGCGCCGGAGCCGTCGCCG**<br>**TGATCAGCGCGGTCCTGCTGGCCCAGCGCTCCAGCGCGCGGGAGTCCCGGGTGGCCCGGAAGTCCGGCCCGC**<br>**CACCGGAACCGGCGGAGGCGGGCACGGAGACGCCCGCGGCCGCCGGGCCCGCCCATCCGGCACCGCCTTCGC**<br>**CGCAGCCCGGTGACCGGCCGGCGGCACGTTCCTCCCAGCCGGTCCCGGAGAGCCGAAAGATCACGGAAAAGG**<br>**AGCAGTCATGACGCGGCCCGTTCTGTTCGATCTCGACGGGACCCTCGTCGACACCCCGAACGCCATCGTCGA**<br>**GAACTTCACCGCCACCCTGGCCGAGCTGGGCGCTCCGGCCGCCTCCCCCGAGGAGATCCGGTCCACCATCGG**<br>**GCTCCCGCTCGAGAAGGCGTTCGCCCAGCTCATCGGAGCCTCCGTGCCCGCCGCCACCCTCGACGCGGCCGT**<br>**GCCGTGCTACCAACGGCTGTACCGCGAACAGATCGTGCCGAAGGCCAAGTCGTTGCTGTACCCCGGAGTCGT**<br>**CGACGGCCTGGCCGAGCTGTCCGAGGCGGGCCTGCTGCTCGCCATCGCCACCAGCAAGCACACCGCGAGTGC**<br>**CGAGGTGCTGCTGACGGCGGCCGGCATCCGGGACCGGTTCGCCATGGTCGTCGGCGCGGACGCCGTCACGGA**<br>**ACCGAAACCGGCTCCGGAGACGGGCCTGCTCATCGCCGCCGAACTGGGCCATCCGGCCGCCCGGGCCATCAT**<br>**GGTCGGTGACACCACCCACGACATCTCGATGGCGCACAACGCCGGTATGCGCAGCGTCGCGGTCACCTACGG**<br>**CATCCACTCGCCTGGCCAACTGGCCGCGGTCCGGCCCACCTGGACGGTGCGGGACTTCCCCACCGCCGTGGA**<br>**GCGGATCCTCCGCGCCGACTGACCACGCCCGACCCCGGCGTGTTCACATTCGAACGGTCTCTGCTTTGACAA**<br>**CATGCTGTGCGGTGTTGTAAAGTCGTGGCCAGGAGAATACGACAGCGTGCAGGACTGGGGAGTTATGCCGT**<br>**GATGCCGTCTCGCACTCCCGCAGGGCACAACAGGGCCGACCACGCCCATGAGTTCACCTCGCACGATCAGCA**<br>**CCAGCCTGCACCGGCGCCCGCCGCCGATCCGGCCGTACCCATCGCAGATCACACACTCGGGGGATCGTCAAC**<br>**GATGAGCACCAGCACGCCGCCACCCGCCACCCGCCAGCACGCCTCCCTGCCCGCCCTGCTGCGCCACCAGGC**<br>**CCGTGAGCGCGGCGACGACACCGCCGTGACAGCGGCGGAGGGCCGTCTCACCTTCGCGGAACTCCACTCCGC**<br>**GGCCACGCGGATGGGCTCCCACCTGGTCGGCGCCGGAGTGTCGCCCGACACCTGCGTCGGGCTCTTCGTGGA**<br>**GCCCTCCGCCGACCTGGTGACAGGTGTGTGGGGCATCCTCACCGCAGGCGCGGCCTATCTTCCGCTCTCGCC**<br>**CGACTACCCGGACTCCCGGCTTCGCCACATGGCGGCGGACGCCGGAGTCGGCGTGGTCGTCACCCAGAGCCA**<br>**CCTGCGCGCCAGGCTGCACGGCCTGGTTCCCGCGGGCACCCTGGTCGTCACCGTGGACGAGGCCACGGACGC**<br>**CGACGGCGCGCACCCCCTTCCCGAGCCCCGGGAGGACCACCTCGCCTACGTCATCCACACCTCCGGCAGCAC**<br>**GGGCAGCCCCAAGGGCGTGATGATCGAACACCGCAGTGTCGTCGCCCAGTTACGGTGGCTGACGCGCCGTGG**<br>**GTATCTCGGCCCGGCGGTCTCCGTCCTGCAGAAGACGCCGATCAGCTTCGACGCGGCGCAGTGGGAGATCCT**<br>**GGCCTGTGCGGCGGGGGCGCGGGTGGTCATGAGTCCGCCGGGGTCCTTCCGCGACCCGCAGGCGGTCATCGA**<br>**CGCGATCCACACGTACGGCGTCACCTGCCTGCAGGGGGTGCCCACCCTGCTGCGGGCCCTCGTGGACACCGG**<br>**TGAGCTGGACACCTGCTCGACACTGACCCGG**<u>AAGCTT</u>(*Hin*dIII)
**sgRNA and donor sequences of pKCcas9::StrepT-switch-d*dptABC***
sgRNA sequences was highlighted in yellow, and donor sequences are in back (upstream homologous arm) or blue (downstream homologous arm). The restriction sites are underlined.
(*Spe*I)<u>ACTAGT</u>TCTCCTCGACTACCTTCGACGTTTTAGAGCTAGAAATAGCAAGTTAAAATAAGGCTAGTCCG<br>TTATCAACTTGAAAAAGTGGCACCGAGTCGGTGCTTTTTTTGAG**GTCGGCCACTTCCGCGCCCGCATCGTCG**<br>**AACCCGGCGGGCACCGTGTTCTGCCGCCCGGCCAGGTCGGCGAGCTGGTCCTCCAGGGAGCCGCCGTCTGCG**<br>**CCGGCTACTGGCAGGCCAAGGAGGAGACCGAGCAGACCTTCGGCCTCACCCTCGACGGCGAGGACGGTCACT**<br>**GGCTGCGCACCGGCGATCTCGCCGCCCTGCACGAAGGGAATCTCCACATCACCGGCCGCTGCAAAGAGGCCC**<br>**TGGTGATACGAGGACGCAATCTGTACCCGCAGGACATCGAGCACGAACTCCGCCTGCAACACCCGGAACTTG**<br>**AGAGCGTCGGCGCCGCGTTCACCGTCCCGGCGGCACCTGGCACGCCGGGCTTGATGGTGGTCCACGAAGTCC**

GCACCCCGGTCCCCGCCGACGACCACCCGGCCCTGGTCAGCGCCCTGCGGGGACGATCAACCGCGAATTCG
GACTCGACGCCCAGGGCATCGCCCTGGTGAGCCGCGGCACCGTACTGCGTACCACCAGCGGCAAGGTCCGCC
GGGGCGCCATGCGTGACCTCTGCCTCCGCGGGGAGCTGAACATCGTCCACGCGGACAAGGGCTGGCACGCCA
TCGCCGGCACGGCCGGAGAGGACATCGCCCCCACTGACCACGCTCCACATCCGCACCCCGCGTAATCGCCGG
AGGGCGGCCCTGCCCTGGAACGGGCACCGCGGTGCCCGCCGACAGCGAGGAGTAGCTCCACATGAACCCGCC
CGAAGCGGTCAGCACGCCCAGCGAGGTCACCGCGTGGATCACCGGACAGATCGCCGAGTTCGTGAACGAGAC
ACCCGACCGGATCGCCGGTGACGCACCCCTGACCGACCATGGCCTCGACTCCGTCTCCGGAGTTGCCCTCTG
CGCGCAGGTCGAGGACCGCTACGGATCGAGGTCGACCCGGAGCTGCTGTGGAGCGTCCCCACACTCAACGA
GTTCGTCCAGGCACTGATGCCCCAGTTGGCCGACCGCGCAGCGCGCGATGGAGGACATACTTCCTCTCACTC
CGCTGCAGGAGGGACTGCTGTTCCACAGTGTTTACGACGAGCAGTCCGTCGACGTGTACACCGTGCAGGTGG
TCGTCGACCTCGAGGGGCCCGTCGACCCCGAAGCACTGCGCGCCGCCGCGGCCGCCCTGCTGCGTCGGCACG
CCAACCTGCGGGCGGCCTTCCGGTACGAGCGGCTGCAGCGCCCCGTGCAGATCATCCCGCGCGAGGTTGCGG
TGCCGTGGGAGCACACCGACGTCGCGAAGCTCGAGGGCGCCGAGCAGAAGGCCGAGATCGAACGCCTGCTGC
ACGACCAGCGGTGGCGCCGCTTCGATCTGACGGCTCCGCCCCTGCTGCGGTTCCTGCTCGTGCGCACAGGCC
ACGACCGGCACCGTTTCGCGCTGACTTTCCATCACATCCTCATGGACGGCTGGTCGATGCCCGTCCTGCTGC
GGGAACTCATCACCCTCTACCGCACCGGCGACGAGACCGCCCTGCCCTGGGTCCGGCCGTACCGGGACTACC
TGGCCTGGATCTCCCGCCGCGACCGGGACGAGGCCGGGCGGGCCTGGTCCAAGGCACTGGCCGGGGTTGACG
AGGCCACCCTCGTCGCCCCGGGTGCCGACCGGGCCGCCGAGCCGCCGCTGTGGACCGAGTCCCGGCTCGAAC
CGGACCTGGCGGCGACGCTCGCCGCCCGCGCCCGCGAGTTCGGCGTCACCCTCAACACCCTCGTCCAGGCCG
CCTGGGCGCTCGTCCTCGGCCGCCTCACCGGCCGCGACGACGTCGTGTTCGGCGTGACCGTGTCCGGCCGGC
CGCCGGAGCTCGCAGGTGTCGAGGACATGGTGGGCCTCTTCATCAACACCGTGCCGCTGCGTGCCGAGCTGC
TGCCGCACGAGAGCCTCCGGGACTTCACCGTCCGCCTCCAGCGCGAACAGATACAGCTCCTCGACCACCAGT
ACGAACGACTGGCGGTCATCCAGCGGCTAAGCTT(*Hin*dIII)

sgRNA sequences of pSET152::StrepT-switch- *dCas9-actI1*

Sequences of N20 are highlighted in yellow, and restriction sites are underlined.

(*Spe*I)ACTAGTTGAGCGCGTGAGGGACCACG**GTTTTAGAGCTAGAAATAGCAAGTTAAAATAAGGCTAGTCCG**
TTATCAACTTGAAAAAGTGGCACCGAGTCGGTGCTTTTTTTGAGTCACCAATAAAAAACGCCCGGCGGCAAC
CGAGCGTTCTGAACAAATCCAGATGGAGTTCTGAGGTCATTACTGGATGTACACCCGAATTC(*Eco*RI)

sgRNA sequences of pSET152::StrepT-switch- *dCas9-ftsZ*

Sequences of N20 are highlighted in yellow, and restriction sites are underlined.

(*Spe*I)ACTAGTAGACCATGTCGGCCCCCTTG**GTTTTAGAGCTAGAAATAGCAAGTTAAAATAAGGCTAGTCCG**
TTATCAACTTGAAAAAGTGGCACCGAGTCGGTGCTTTTTTTGAGTCACCAATAAAAAACGCCCGGCGGCAAC
CGAGCGTTCTGAACAAATCCAGATGGAGTTCTGAGGTCATTACTGGATGTACACCCGAATTC(*Eco*RI)

Supplementary References

1. Chater,K.F. and Wilde,L.C. (1980) *Streptomyces albus* G mutants defective in the *Sal*GI restriction-modification system. *J. Gen. Microbiol.*, **116**, 323-334.
2. Wang,X., Fu,Y., Wang,M. and Niu,G. (2021) Synthetic cellobiose-inducible regulatory systems allow tight and dynamic controls of gene expression in *Streptomyces*. *ACS Synth. Biol.*, **10**, 1956-1965.
3. Kieser,T., Bibb,M.J., Buttner,M.J., Chater,K.F. and Hopwood,D. (2000) Practical *Streptomyces* Genetics, John Innes Foundation, Norwich, UK.
4. Gomez-Escribano,J.P. and Bibb,M.J. (2011) Engineering *Streptomyces coelicolor* for heterologous expression of secondary metabolite gene clusters. *Microb. Biotechnol.*, **4**, 207-215.
5. Cruz-Morales,P., Vijgenboom,E., Iruegas-Bocardo,F., Girard,G., Yáñez-Guerra,L.A., Ramos-Aboites,H.E., Pernodet,JL., Anné,J., van Wezel,G.P. and Barona-Gómez,F. (2013) The genome sequence of *Streptomyces lividans* 66 reveals a novel tRNA-dependent peptide biosynthetic system within a metal-related genomic island. *Genome Biol. Evol.*, **5**, 1165-1175.
6. Jiang,L., Wang,L., Zhang,J., Liu,H., Hong,B., Tan,H. and Niu,G. (2015) Identification of novel mureidomycin analogues via rational activation of a cryptic gene cluster in *Streptomyces roseosporus* NRRL 15998. *Sci. Rep.*, **5**, 14111.
7. Paget,M.S., Chamberlin,L., Atrih,A., Foster,S.J. and Buttner,M.J. (1999) Evidence that the extracytoplasmic function sigma factor σ^E is required for normal cell wall structure in *Streptomyces coelicolor* A3(2). *J. Bacteriol.*, **181**, 204-211.
8. Datsenko,K.A. and Wanner,B.L. (2000) One-step inactivation of chromosomal genes in *Escherichia coli* K-12 using PCR products. *Proc. Natl. Acad. Sci. U.S.A.*, **97**, 6640-6645.

9. Gust,B., Challis,G.L., Fowler,K., Kieser,T. and Chater,K.F. (2003) PCR-targeted *Streptomyces* gene replacement identifies a protein domain needed for biosynthesis of the sesquiterpene soil odor geosmin. *Proc. Natl. Acad. Sci. U.S.A.*, **100**, 1541-1546.

10. Li,R., Xie,Z., Tian,Y., Yang,H., Chen,W., You,D., Liu,G., Deng,Z. and Tan,H. (2009) *polR*, a pathway-specific transcriptional regulatory gene, positively controls polyoxin biosynthesis in *Streptomyces cacaoi* subsp. *asoensis*. *Microbiology,* **155**, 1819-1831.

11. Sun,J., Kelemen,G.H., Fernández-Abalos,J.M. and Bibb,M.J. (1999) Green fluorescent protein as a reporter for spatial and temporal gene expression in *Streptomyces coelicolor* A3(2). *Microbiology*, **145**, 2221-2227.

12. Bierman,M., Logan,R., O'Brien,K., Seno,E.T., Rao,R.N. and Schoner,B.E. (1992) Plasmid cloning vectors for the conjugal transfer of DNA from *Escherichia coli* to *Streptomyces* spp. *Gene.*, **116**, 43-49.

13. Du,D., Zhu,Y., Wei,J., Tian,Y., Niu,G. and Tan,H. (2013) Improvement of gougerotin and nikkomycin production by engineering their biosynthetic gene clusters. *Appl. Microbiol. Biotechnol.*, **97**, 6383-6396.

14. Li,J., Li,Y., Niu,G., Guo,H., Qiu,Y., Lin,Z., Liu,W. and Tan,H. (2018) NosP-regulated nosiheptide production responds to both peptidyl and small-molecule ligands derived from the precursor peptide. *Cell Chem. Biol.*, **25**, 143-153.

15. Zhao,Y., Li,L., Zheng,G., Jiang,W., Deng,Z., Wang,Z. and Lu,Y. (2018) CRISPR/dCas9-mediated multiplex gene repression in *Streptomyces*. *Biotechnol. J.*, **13**, e1800121.

16. Huang,H., Zheng,G., Jiang,W., Hu,H. and Lu,Y. (2015) One-step high-efficiency CRISPR/Cas9-mediated genome editing in *Streptomyces*. *Acta Biochim. Biophys. Sin.*, **47**, 231-243.

17. Pullan,S.T., Chandra,G., Bibb,M.J. and Merrick,M. (2011) Genome-wide analysis of the role of GlnR in *Streptomyces venezuelae* provides new insights into global nitrogen regulation in actinomycetes. *BMC Genomics*, **12**, 175.
